# CD171- and GD2-specific CAR-T cells potently target retinoblastoma cells in preclinical in vitro testing

**DOI:** 10.1186/s12885-019-6131-1

**Published:** 2019-09-09

**Authors:** Lena Andersch, Josefine Radke, Anika Klaus, Silke Schwiebert, Annika Winkler, Elisa Schumann, Laura Grunewald, Felix Zirngibl, Carina Flemmig, Michael C. Jensen, Claudia Rossig, Antonia Joussen, Anton Henssen, Angelika Eggert, Johannes H. Schulte, Annette Künkele

**Affiliations:** 1 Department of Pediatric Oncology and Hematology, Charité – Universitätsmedizin Berlin, corporate member of Freie Universität Berlin, Humboldt-Universität zu Berlin, and Berlin Institute of Health, Augustenburger Platz 1, 13353 Berlin, Germany; 2 Department of Neuropathology, Charitéplatz 1, Charité, Universitätsmedizin Berlin, 10117 Berlin, Germany; 3 Berlin Institute of Health (BIH), Anna-Louisa-Karsch-Str. 2, 10178 Berlin, Germany; 4 German Cancer Consortium (DKTK), Partner Site Berlin, CCCC (Campus Mitte), Invalidenstr. 80, 10115 Berlin, Germany; 5 Ben Towne Center for Childhood Cancer Research, Seattle Children’s Research Institute, Seattle, WA USA; 6 Seattle Children’s Research Institute, Seattle, WA USA; 7 Fred Hutchinson Cancer Research Center, Seattle, WA USA; 8 Department of Bioengineering, University of Washington, Seattle, WA USA; 9 Department of Pediatric Hematology and Oncology, University Children’s Hospital Muenster, Albert-Schweitzer-Campus 1, 48149 Muenster, Germany; 10 Department of Ophthalmology, Charité – Universitätsmedizin Berlin, Hindenburgdamm 30, 12200 Berlin, Germany; 11 German Cancer Research Center (DKFZ), Im Neuenheimer Feld 280, 69120 Heidelberg, Germany

**Keywords:** Adoptive T-cell immunotherapy, Retinoblastoma, CD171, GD2, Antigen loss, Sequential CAR-T cell therapy

## Abstract

**Background:**

Chimeric antigen receptor (CAR)-based T cell therapy is in early clinical trials to target the neuroectodermal tumor, neuroblastoma. No preclinical or clinical efficacy data are available for retinoblastoma to date. Whereas unilateral intraocular retinoblastoma is cured by enucleation of the eye, infiltration of the optic nerve indicates potential diffuse scattering and tumor spread leading to a major therapeutic challenge. CAR-T cell therapy could improve the currently limited therapeutic strategies for metastasized retinoblastoma by simultaneously killing both primary tumor and metastasizing malignant cells and by reducing chemotherapy-related late effects.

**Methods:**

CD171 and GD2 expression was flow cytometrically analyzed in 11 retinoblastoma cell lines. CD171 expression and T cell infiltration (CD3^+^) was immunohistochemically assessed in retrospectively collected primary retinoblastomas. The efficacy of CAR-T cells targeting the CD171 and GD2 tumor-associated antigens was preclinically tested against three antigen-expressing retinoblastoma cell lines. CAR-T cell activation and exhaustion were assessed by cytokine release assays and flow cytometric detection of cell surface markers, and killing ability was assessed in cytotoxic assays. CAR constructs harboring different extracellular spacer lengths (short/long) and intracellular co-stimulatory domains (CD28/4-1BB) were compared to select the most potent constructs.

**Results:**

All retinoblastoma cell lines investigated expressed CD171 and GD2. CD171 was expressed in 15/30 primary retinoblastomas. Retinoblastoma cell encounter strongly activated both CD171-specific and GD2-specific CAR-T cells. Targeting either CD171 or GD2 effectively killed all retinoblastoma cell lines examined. Similar activation and killing ability for either target was achieved by all CAR constructs irrespective of the length of the extracellular spacers and the co-stimulatory domain. Cell lines differentially lost tumor antigen expression upon CAR-T cell encounter, with CD171 being completely lost by all tested cell lines and GD2 further down-regulated in cell lines expressing low GD2 levels before CAR-T cell challenge. Alternating the CAR-T cell target in sequential challenges enhanced retinoblastoma cell killing.

**Conclusion:**

Both CD171 and GD2 are effective targets on human retinoblastoma cell lines, and CAR-T cell therapy is highly effective against retinoblastoma in vitro. Targeting of two different antigens by sequential CAR-T cell applications enhanced tumor cell killing and preempted tumor antigen loss in preclinical testing.

**Supplementary information:**

**Supplementary information** accompanies this paper at 10.1186/s12885-019-6131-1.

## Background

Children suffering from unilateral intraocular retinoblastoma can be cured with the enucleation of the eye without any further treatment. However, 45% of all reported cases encompass the heritable form of retinoblastoma, a biallelic germline mutation of the retinoblastoma tumor suppressor gene *RB1.* [1] In 80% of children with heritable disease, retinoblastoma affects both eyes (bilateral) and 5% of the cases are associated with an intracranial tumor (trilateral). [2]

Saving life is the highest goal in retinoblastoma therapy followed by vision salvage. In order to salvage vision, if reasonable, the eye is preserved in case of localized tumors, which are treated with laser application cryo- or brachytherapy and/or local intra-arterial chemotherapy. In large tumors, initial reduction of the tumor size can be achieved by systemic chemotherapy, which enables subsequent local treatment options. High-dose systemic chemotherapy with stem cell rescue is reserved for non-responsive extraocular and/or metastastic disease. [3, 4] Overall survival is high in western countries (> 95%). However, due to a higher rate of secondary malignancies, long-term overall survival is reduced in children treated with eye preserving radio- and/or chemotherapy compared with enucleation alone. [5, 6]

Retinoblastoma can disseminate through the optic nerve into the central nervous system and through the sclera via lymphatic or blood circulation of the orbit bones to distant metastatic sites in the lymph nodes, bones, bone marrow and liver. [7] In these cases, salvage with high-dose chemotherapy is often not successful. In addition, high-dose chemotherapy is highly aggressive, and can create lifelong sequelae and morbidity for the patient. [4, 7–9] Therefore, the search for more efficient and better tolerated treatment options is warranted.

Adoptive T cell therapy might be a promising alternative. Adoptive T cell immunotherapy, in which T lymphocytes isolated from patients are engineered to express CD19-specific chimeric antigen receptors (CARs), has shown striking anti-tumor effects against acute B cell leukemia and non-Hodgkin lymphoma. [10–13] CAR-T cells combine two striking characteristics of the immune system: the exquisite antigen-binding specificity of a monoclonal antibody and the potent toxicity of cytotoxic T lymphocytes. A spacer domain connects the antigen-binding domain, commonly a single-chain variable fragment (scFv) of a monoclonal antibody, to the transmembrane domain followed by a T cell signaling module. [14] Spacer length influences CAR-T cell function, as the distance between the CAR-T cell and tumor antigen epitope must be uniquely adjusted for optimal bridging. [15, 16] The signaling module incorporates the CD3-zeta domain and a co-stimulatory domain, commonly either 4-1BB or CD28, to provide signals necessary for full T cell activation. The co-stimulatory domain used can affect CAR-T cell functionality by triggering different signaling pathways. The 4-1BB domain has been associated with increased CAR-T cell persistence [17], but the CD28 domain has been demonstrated to enhance CAR-T cell cytotoxicity. [18]

GD2 and CD171 may present promising targets for CAR-T cell therapy of retinoblastoma. The GD2 ganglioside is expressed on the cell surface of several neuroectodermal tumors, including retinoblastoma. [19–22] GD2 expression is highly restricted in nonmalignant tissue with only low-level expression on peripheral nerves, skin melanocytes, brain and osteoprogenitors. [23, 24] Anti-GD2 monoclonal antibodies have already proven safety and efficacy in clinical trials and are included in the standard treatment for children with high-risk neuroblastoma demonstrating its role as a target for immunotherapy. [25–27] CD171 (formerly L1CAM) plays a crucial role during nervous system development, including neuronal migration and axon guidance. [28] It was recently shown to be expressed in retinoblastomas, and expression in the Y79 and Rb1 cell lines correlated with increased in vitro proliferation and chemoresistance in a mouse model. [29] In most tumor entities CD171 expression is further described to be associated with poor prognosis making it a potential target for new treatment options like immunotherapy. [30–32] CD171 expression by normal tissue was examined by our group and a safety study in non-human primates revealed no on-target, off-tumor toxicity after infusion of up to 1 × 10^8^/kg CD171-specific CAR-T cells in non-conditioned animals. [33] CAR-T-cell therapy could represent a new treatment option for extraocular and/or metastasized retinoblastoma. If successful, CAR-T cell therapy could also be integrated with vision-preserving therapies for children with bilateral retinoblastoma to reduce therapeutic toxicity and late treatment-related effects such as secondary cancers, while preserving vision in at least one eye.

As a first step towards a CAR-T-cell therapy targeting retinoblastoma, we here assessed CD171 expression in retrospective retinoblastoma samples and investigated the killing efficacy of CD171- and GD2-specific CAR-T cells harboring different spacer lengths (short versus long) and intracellular co-stimulatory domains (CD28 versus 4-1BB) in a panel of three different cell lines. Our aim was to assess functional differences of various CAR-T cell constructs to select the most efficient for further preclinical testing and entry into the clinic trials.

## Methods

### Retinoblastoma samples and cell lines

Tumor samples and medical records were retrospectively evaluated from 30 children diagnosed with retinoblastoma between January 2003 and August 2013 in the Department of Pediatric Oncology and Hematology, *Charité - Universitätsmedizin Berlin*. Cell lines RB247C3, RB355, RB383, RB522, RB1021 and RB3823 were kindly provided by Brenda Gallie (Department of Ophthalmology and Vision Sciences, Hospital for Sick Children, Toronto, Ontario, Canada). [34] RBL13, RBL15 and RBL30 cell lines were established at the University Hospital Essen and were obtained from the Institute of Cell Biology, University Hospital Essen. [35] Cell lines, Y79 (DSMZ-ACC 246), WERI-Rb1 (DSMZ-ACC 90) and NALM-6 (DSMZ-ACC 128), were purchased from the German collection of microorganisms and cell cultures. The WERI-Rb1, RBL15 and RB355 retinoblastoma cell lines and the NALM-6 B cell leukemia cell line were lentivirally transduced with green fluorescent protein (GFP)-firefly luciferase (ffLuc)_epHIV7, and GFP-expressing cells were selected using fluorescence-activated cell sorting (FACS). WERI-Rb1 and NALM-6 cells were maintained in RPMI 1640 (Gibco Life technologies) supplemented with 15% heat-inactivated fetal calf serum (FCS, Sigma-Aldrich). RBL15 and RB355 cell lines were maintained in DMEM (Gibco Life technologies) supplemented with 10% heat-inactivated FCS, 10 μg/ml insulin (Sigma-Aldrich) and 50 μM 2-mercaptoethanol (Sigma-Aldrich). The TMLCL EBV-transformed lymphoblastoid cell line was maintained in RPMI 1640 supplemented with 10% heat-inactivated FCS and 2 mM L-glutamine (Biochrom).

### Retinoblastoma immunohistochemistry

The CD171 and CD3 (T cell marker) were immunohistochemically detected in tumor sections. Primary antibodies were omitted as negative controls and kidney tissue (CD171) was used as positive control (see Additional file 1: Figure S1). T cell infiltration (number of CD3^+^ lymphocytes per cm^2^) in preserved retinoblastoma tissue sections was quantified using the Stereo Investigator® system (MBF Bioscience). Methods of immunohistochemistry are described in an additional file in more detail (see Additional file 2).

### CAR constructs

The CD171-specific CE7-CAR [36] and GD2-specific 14.2GA-CAR [37] were previously described, and were cloned into the SIN epHIV7 lentiviral vector plasmid. CAR lentiviruses were propagated in 293 T cells. [38] The scFvs in both CAR constructs were codon optimized and subsequently linked to a 12 (short) or 229 (long) amino acid spacer domains from the human IgG4 hinge. The long spacer domain was modified by substituting L235D and N297Q to reduce binding to the IgG Fc gamma receptor. [39] The spacer domain connects the antigen-binding domain to CD28 transmembrane domain followed by the signaling module containing the CD3zeta cytoplasmic domain and either 4-1BB or CD28. The CAR constructs were linked downstream to a T2A self-cleaving peptide and truncated epidermal growth factor receptor (EGFRt) allowing CAR-T cell detection and enrichment.

### Generation and culture of CD171- and GD2-specific CAR-T cells

Apheresis products were obtained from healthy donors and peripheral blood mononuclear cells were isolated using Ficoll-Paque (GE Healthcare). CD8^+^ T cells were obtained by positive selection using immunomagnetic microbeads (Miltenyi Biotec), and activated with anti-CD3/CD28 beads (Life Technologies). On day three, activated CD8^+^ T cells were transduced with the CAR containing lentivirus. The EGFRt^+^ CAR-T cell subset was enriched by immunomagnetic selection with biotin-conjugated cetuximab (Bristol-Myers Squibb) and streptavidin microbeads (Miltenyi Biotec). T cells used as mock negative controls alongside CAR-T cells in experiments were not lentivirally transduced. CAR and mock control T cells were stimulated with irradiated peripheral blood mononuclear cells, irradiated TMLCL, and OKT3 (30 ng/mL, Miltenyi Biotec), expanded according to a rapid expansion protocol [36] and cryopreserved until further use. Cryopreserved cells were thawed, expanded as described above and functional in vitro assays were conducted between days 11 and 16 of culture.

### Functional assays

For cytokine release assays, 2 × 10^5^ T cells were seeded together with stimulator cells at a 2:1 effector:target ratio. All data points were performed as technical triplicates. After 24 h, supernatants were collected and stored at − 80 °C until analysis of IFNG and IL2 using the OptEIA™ Set (BD Biosciences) enzyme-linked immunosorbent assay (ELISA) kits in accordance with the manufacturer’s instructions. CAR-T cell-induced cytotoxicity was quantified in a biophotonic luciferase assay in which the retinoblastoma cells, stably transduced with a GFP-ffLuc_epHIV7 reporter, served as tumor target cells. Target cells were co-cultured in triplicate with mock-transduced or CAR-T cells. The maximal biophotonic luciferase signal was defined by target cells plated alone at the same densities (RLU_max,_ maximal relative light unit). After 24, 48 or 72 h, 0.14 mg D-luciferin (PerkinElmer Inc.)/ml medium was added to each well, and the biophotonic signal detected. Lysis was determined as [1-(RLU_sample_/RLU_max_)]× 100 in relation to untreated cells. For sequential treatment, the additive amount of tumor lysis was calculated related to viable tumor cells at day 3.

### Flow cytometric marker and antigen detection

Cell-surface expression of GD2 (cat#565991, BD Biosciences), CD8 (cat#301041, BioLegend) and CD171 (cat#130–100-691, Miltenyi Biotec) was detected by fluorophore-conjugated monoclonal antibodies. EGFRt expression was detected using biotinylated cetuximab (Bristol-Myers Squibb) and a phycoerythrin (PE)-conjugated streptavidin antibody (cat#12–4317-87, BioLegend). Activation and exhaustion were assessed by fluorophore-conjugated monoclonal antibodies detecting CD137 (cat#309819, BioLegend), CD25 (cat#302622, BioLegend), PD1 (also known as PDCD1 or CD279, cat#329922, BioLegend), TIM3 (cat#345006, Biolegend) and LAG3 (cat#565721, BD Biosciences). Flow cytometry was performed on a Fortessa X-20 (BD Biosciences) and data processed using FlowJo software (Tree Star Inc.). Dead cells were excluded from analyses using LIVE/DEAD™ Fixable Green Dead Cell Stain Kit (cat#L23101, Life Technologies). QuantiBRITE PE calibration beads (BD Biosciences) were used to determine GD2 and CD171 antigen density on retinoblastoma cells according to the manufacturer’s instructions.

### Statistical analysis

To determine significance of differences in cytotoxic activity and cytokine release of GD2-and CD171-specific CAR-T cells compared with negative control mock T-cells, unpaired Student’s t-test was performed using GraphPad prism (GraphPad Software). *P* values < 0.05 were considered statistically significant.

## Results

### CD171 expressed in primary retinoblastomas and retinoblastoma cell lines

**Fig. 1:**
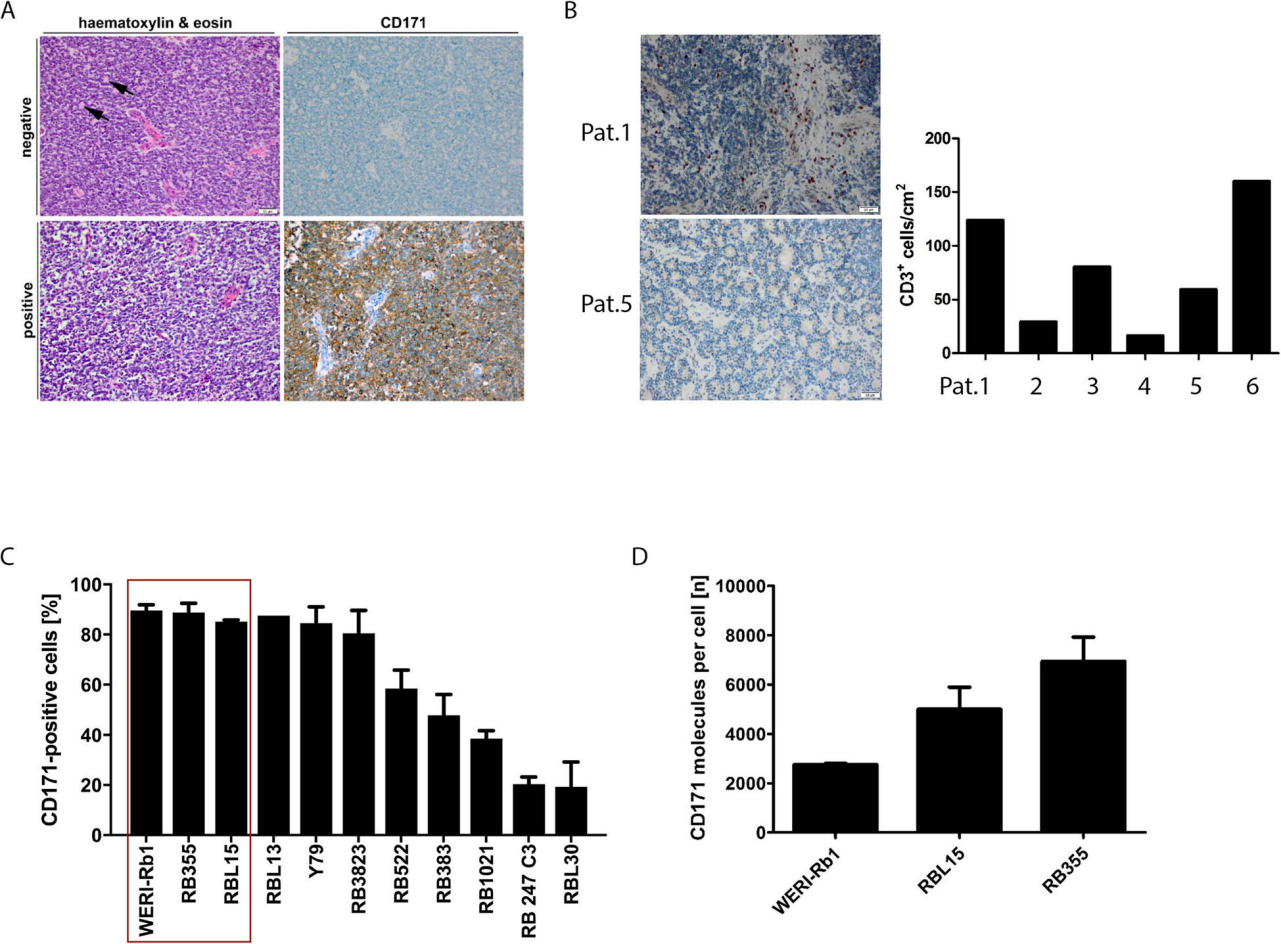
CD171 is expressed in retinoblastomas and cell lines derived from retinoblastomas. **a** The tumor is composed of small undifferentiated, blue cells (hematoxylin & eosin stain). Flexner-Wintersteiner rosettes may be found and are highly characteristic (arrows). The expression of CD171 was classified as negative or positive. (Scale bar: 50 μm). **b** CD3^+^-staining is exemplarily shown for patient 1 and patient 5. CD3^+^ infiltration in 6 of 30 randomly chosen primary retinoblastoma tumor samples. **c** CD171 cell surface expression on eleven established retinoblastoma cell lines detected with antihuman fluorochrome-conjugated CD171 antibody (*n* = 3). **d** Number of CD171 molecules per cell calculated based on a PE-bead based assay for three selected cell lines, WERI-Rb1, RBL15 and RB355 (mean ± SD, n = 3)

To investigate the potential of immunotherapeutically targeting retinoblastoma via the tumor antigen, CD171, we immunohistochemically evaluated its presence and abundance in 30 primary retinoblastoma samples. Tumor samples with ≥5.0% of cells expressing CD171 were considered CD171-positive as was previously described by Jo et al. [29] Half of the analyzed retinoblastomas (15/30) expressed CD171 in various frequencies (Fig. 1a, Additional file 3: Figure S2). Analysis of respective clinical patient data confirmed that a representative patient cohort was investigated (Table 1). Median age at diagnosis was 2.1 (range 0.12–6.5) years, and the cohort included 15 (50%) boys and 15 (50%) girls. Most tumors from this patient group were classified as ICRB group D (57%). T cell infiltration was assessed in 6 retinoblastomas randomly selected from our cohort of 30 retinoblastomas using immunohistochemical detection of the T cell marker, CD3 (Fig. 1b). All analyzed retinoblastomas were infiltrated by T cells demonstrating potential access to retinoblastoma for T cells, and infiltration (quantification of CD3^+^ cells) ranged from 16.3 CD3^+^ cells/cm^2^ to 160.1 CD3^+^ cells/cm^2^. We next assessed CD171 expression in 11 retinoblastoma cell lines by flow cytometry. CD171 was expressed on almost all cells (80–90%) in 6/11 of the retinoblastoma cell lines analyzed, with 3 cell lines moderately expressing CD171 (39–58%) and 2 cell lines exhibiting low-level (< 20%) expression (Fig. 1c). We selected the 3 cell lines (WERI-Rb1, RBL15 and RB355) that most homogenously expressed CD171 for use in experiments to preclinically evaluate CD171-specific CAR-T cell therapy. To assure adequate target expression density on these 3 cell lines, the QuantiBRITE quantification method was applied. Highest antigen density was detected in RB355 cells (mean = 6932 molecules/cell), with RBL15 (mean = 4988 molecules/cell) and WERI-Rb1 (mean = 2757 molecules/cell) cells with slightly lower but substantial target densities (Fig. 1d). These data reveal CD171 as possible target for retinoblastoma-specific CAR-T cell therapy.

**Table 1:** Patient and tumor characteristics included in primary retinoblastoma cohort

Patients	Total	CD171 positive*	CD171 negative
N (%)	30 (100)	15 (50)	15 (50)
Minimum age at diagnosis (years)	0.1	0.4	0.1
Maximum age at diagnosis (years)	6.5	6.4	6.5
Median age at diagnosis (years)	2.1 (0.12–6.5)	1.8	2.1
Sex (%)	♀: 15 (50)	♀:9 (60)	♀:6 (40)
	♂: 15 (50)	♂:6 (40)	♂:9 (60)
Unilateral (%)	23 (77)	12 (80)	11 (73)
Bilateral (%)	7 (23)	3 (20)	4 (27)
ICRB (%)			
A	0 (0)	0 (0)	0 (0)
B	0 (0)	0 (0)	0 (0)
C	1 (3)	1 (7)	0 (0)
D	17 (57)	9 (60)	7 (47)
E	7 (20)	3 (20)	4 (26)
n.a.	6 (20)	2 (13)	4 (26)

* ≥ 5% CD171 positive cells, N = number, ICRB = International Classification of Retinoblastoma

### CD171-specific CAR-T cells target and kill retinoblastoma cells but induce escape mechanisms

**Fig. 2:**
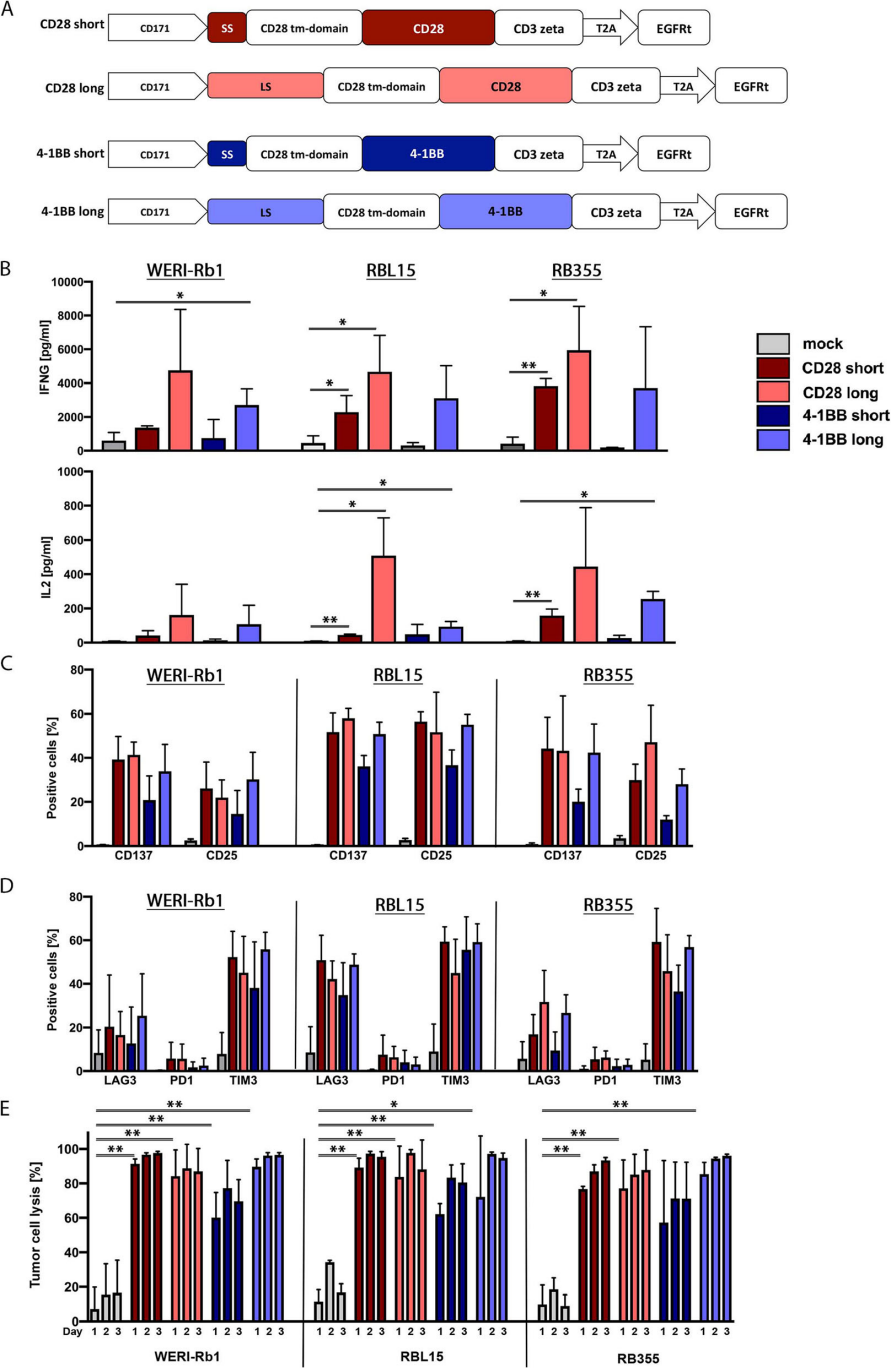
CD171-specific CAR-T cells recognize and kill retinoblastoma cell lines in vitro. **a** Scheme of CD171-specific 2nd generation CAR variants displaying color-coding of the different constructs used. **b** IFNG and IL2 release of CAR-T cells following a co-culture at a 2:1 effector:target (E:T) ratio with retinoblastoma cell lines (mean ± SD, n = 3). Cell surface expression of activation markers CD137 and CD25 **c** and inhibitory receptors LAG3, PD1 and TIM3 (**d**) on CAR-T cells following a 24-h tumor co-culture at a 2:1 E:T ratio (mean ± SD, n = 3). Color-coding is the same as used in B. **e** Cytotoxicity of CD171-specific CAR-T cells is determined by luciferase-based killing assay following a tumor co-culture for 24, 48 and 72 h at a 2:1 E:T ratio (mean ± SD, n = 3). Color-coding is the same as used in B. * *p* ≤ 0.5; **, *p* ≤ 0.01

Potential CAR-T cell efficacy against the selected retinoblastoma cell lines was assessed using in vitro assays. We first assessed the relative ability of co-cultured retinoblastoma cells to activate and induce cytokine release by T cells harboring different CD171-specific CAR constructs. CD8^+^ bulk cells from healthy donors were lentivirally transduced with 4 different CAR constructs containing either a short or long spacer domain combined with either a 4-1BB or CD28 co-stimulatory domain (Fig. 2a). The lentiviral vector also included a truncated EGF receptor without the signaling domain (EGFRt), used to assess transduction efficacy. CAR-expressing cells were enriched for EGFRt expression by immunomagnetic positive selection [27], yielding T cell populations with 86.7–94.6% of cells expressing each CAR construct (see Additional file 4: Figure S3A). Cytokine release (IFNG and IL2) from CAR-T cells following co-culture with WERI-Rb1, RBL15 or RB355 cell lines was quantified by ELISA. Encounter with any of the 3 retinoblastoma cell lines induced IFNG and IL2 release from CD171-specific CAR-T cells but not mock-transduced T cells used as negative controls (Fig. 2b). Similarly, co-culture with the CD171-negative B cell leukemia cell line, NALM-6, did not result in any cytokine release, proving CAR-T cell specificity for CD171 (Additional file 5: Figure S4A + B). Activation markers (CD25 and CD137) on T cells harboring the CD171-specific CAR constructs were also assessed by FACS analysis after retinoblastoma cell encounter. Any of the 3 retinoblastoma cells lines tested also induced both activation markers in co-cultured CD171-specific CAR-T cells (Fig. 2c). The RBL15 cell line was more capable of activating CAR-T cells compared to the other 2 cell lines. Since CAR-T cell activation is regulated by both positive and negative signals provided by the co-stimulatory domain, expression of the PD1, TIM3 and LAG3 inhibitory receptors after retinoblastoma encounter was also assessed in FACS analyses. Co-culture with any of the 3 retinoblastoma cell lines consistently induced TIM3 expression in approximately half the T cell population, while PD1 expression remained below 15% in every case excluding an inhibitory impact on CAR-T cell function by the PD1/PDL1-axis in our in vitro model (Fig. 2d). Induction of LAG3 expression was moderate and varied with retinoblastoma cell type. Our results demonstrate that exposure to retinoblastoma cells activated CD171-specific CAR-T cells and induced cytokine release.

Having demonstrated CAR-T cell activation after retinoblastoma encounter, we investigated the ability of CAR-T cells to kill retinoblastoma cells in a luciferase-based reporter assay. The WERI-Rb1, RBL15 and RB355 cell lines were transduced with a GFP-firefly luciferase reporter plasmid to support viable tumor cell quantification. T cells harboring the various CD171-specific constructs were co-cultured with retinoblastoma reporter cells in a 2:1 effector:target ratio. Cytotoxicity was assessed following 24 h, 48 h and 72 h of co-culture by measuring the biophotonic signal released by the remaining viable tumor cells. CD171-specific CAR-T cells killed significantly more retinoblastoma cells, regardless of cell type, than mock-transduced negative control T cells (Fig. 2e). There was no killing of the CD171 negative cell line, NALM-6 (Additional file 5: Figure S4D). Further, CD171-specific CAR-T cells showed a dose-dependent cytotoxicity of retinoblastoma cells. Cytotoxicity ranged from 97% with a 5:1 effector:target ratio to 76% (1:1 effector:target ratio) to no killing (1:10 effector:target ratio; Additional file 6: Figure S5). These experiments demonstrate that CAR-T cells targeting CD171 efficiently kill retinoblastoma cells in vitro.

Since it is known that different spacers and co-stimulatory domains have influence on the performance of CAR-T cells we compared the T cells harboring different CAR constructs in terms of cytokine release, activation, expression of inhibitory receptors and killing. T cells harboring the CD171 CAR construct with the long spacer and CD28 co-stimulatory domain (CD171-long-CD28) released the most IFNG and IL2 after encounters with any tested cell line (Fig. 2b), although differences did not reach any statistically significance. All CAR-T cells were comparably well activated following co-culture with WERI-Rb1, RBL15 or RB355 cell lines, except T cells harboring the CD171-short-4-1BB construct which displayed the lowest levels of both markers (Fig. 2c). Co-culture with any of the 3 retinoblastoma cell lines consistently induced TIM3 expression, regardless of the CD171-specific CAR construct type expressed (Fig. 2d). The CAR construct used did not have as large an impact on the CAR-T cell ability to kill retinoblastoma cells as it had on the cytokine release by the co-cultured CAR-T cells. Most CD171-specific CAR-T cells were able to kill nearly 100% (Fig. 2e) of the retinoblastoma cells underling the potency of CAR-T cell therapy against retinoblastoma. As already described by Long et al., the ability of CAR-T cells to produce polyfunctional cytokines in response to antigen encounter is a better predictor of CAR-T cell antitumor efficacy in vivo than cytolytic efficacy alone. [40] Since the CAR construct utilizing the long spacer and CD28 co-stimulatory domains produced slightly higher amounts of IFNG and IL2 in vitro*,* it was selected for the remaining experiments.

**Fig. 3:**
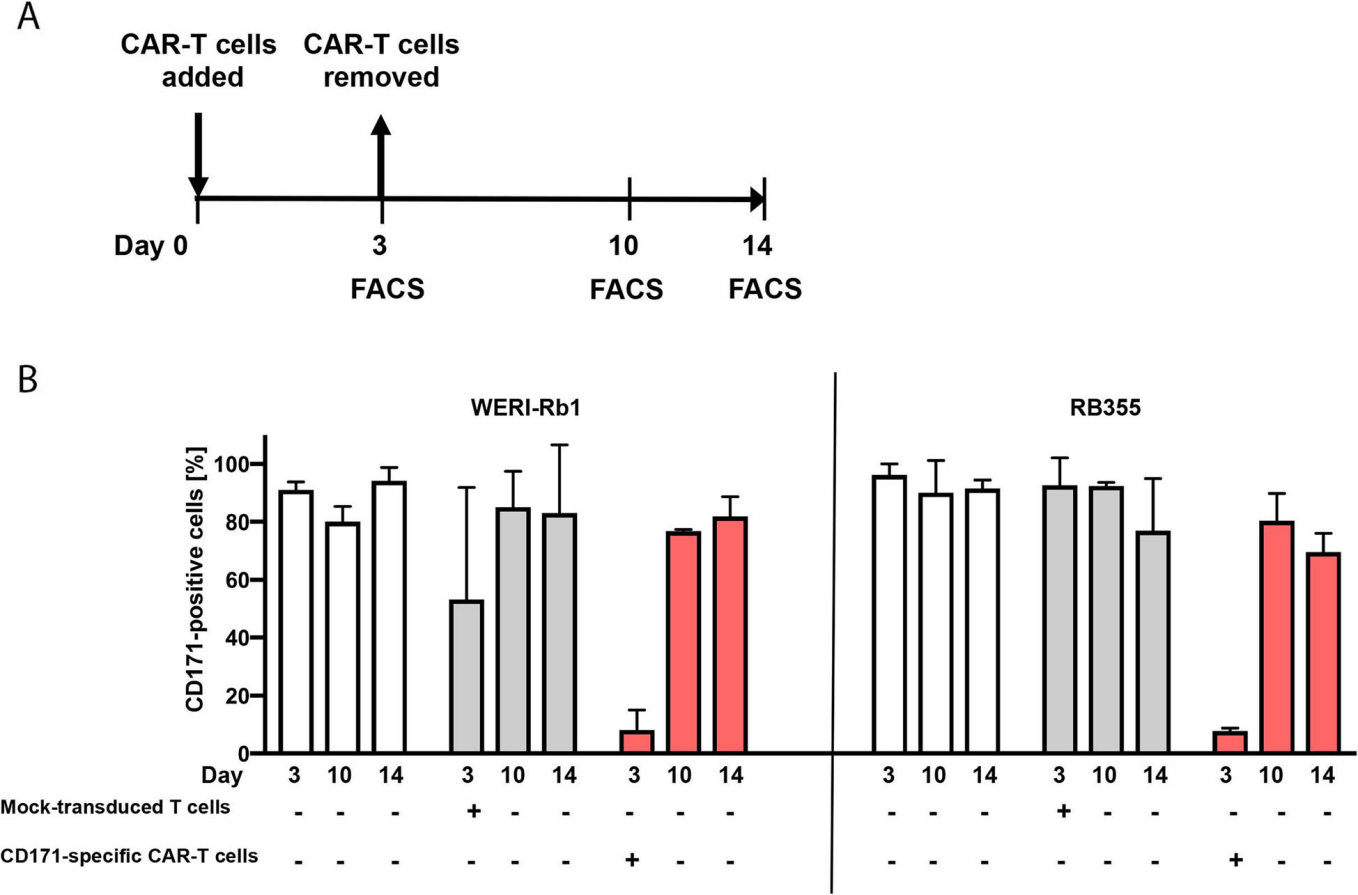
CD171-specific CAR-T cell treatment leads to transient antigen loss. **a** Scheme of the experimental process. Time-dependent cell surface expression of CD171 on WERI-Rb1 (**b**) and RB355 cells (**c**) following 3-day co-culture with CD28-long spacer CAR-T cells and on day 10 and 14 after CAR-T cells removal (mean ± SD, n = 3)

Tumor cells use different strategies to escape attacks from the immune system. One strategy is to downregulate the antigens being targeted by host T cells or the engineered CAR-T cells. [41] To explore whether retinoblastoma cells were utilizing this strategy, we investigated CD171 expression on WERI-Rb1 and RB355 cells via FACS analysis at several time points after transient co-culture with CD171-specific CAR-T cells (Fig. 3a). Transient encounter with CD171-specific CAR-T cells initially reduced CD171 expression on WERI-Rb1 and RB355 cells by 11.4- and 12.3-fold, respectively when assessed on day 3 concomitantly with CAR-T cell removal (Fig. 3b, Additional file 7: Figure S6). However, CD171 expression returned on retinoblastoma cells by day 10, with antigen levels comparable to untreated cells, and remained at these levels on day 14. The differentially regulated antigen expression indicates the potential immune evasion of retinoblastoma cell lines after CAR-T cell encounter revealing the need of targeting more than 1 antigen.

### GD2 is a second promising target for CAR-T cell therapy in retinoblastoma

**Fig. 4:**
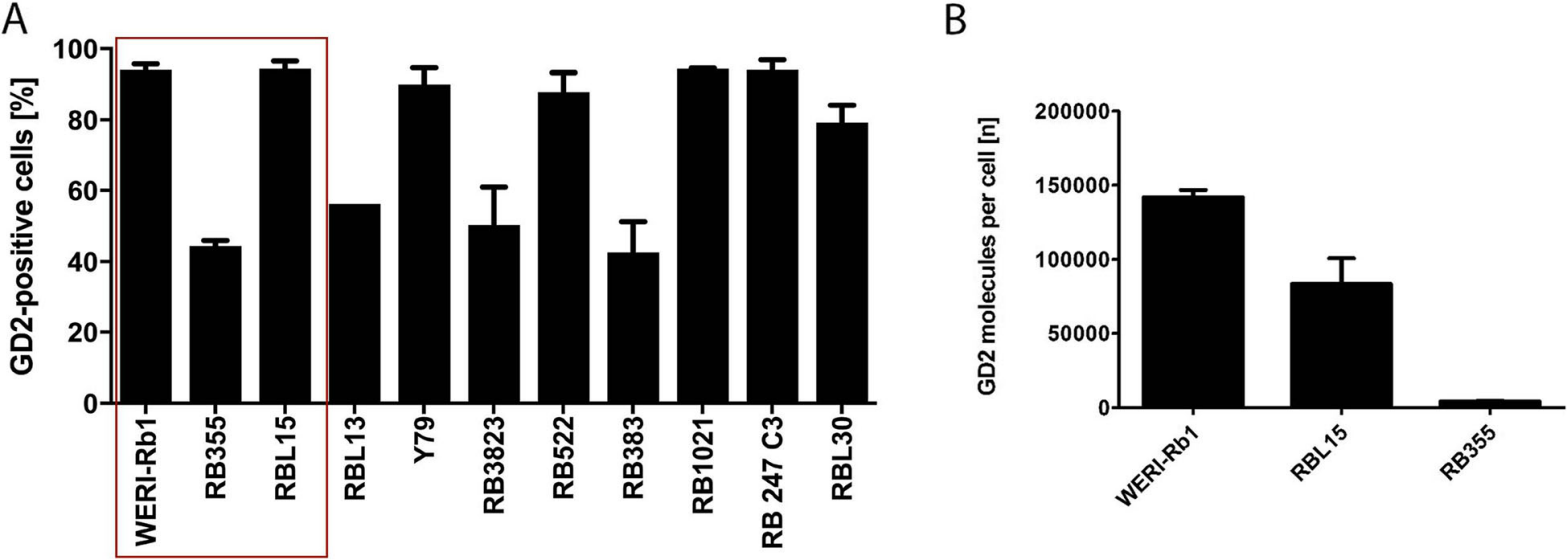
GD2 is expressed on retinoblastoma cell lines. **a** GD2 cell surface expression on eleven established retinoblastoma cell lines detected with antihuman fluorochrome-conjugated GD2 antibody. **b** Number of GD2 molecules per cell calculated based on a PE-bead based assay for 3 selected cell lines, WERI-Rb1, RBL15 and RB355 (mean ± SD, n = 3)

We explored whether GD2 could be effective as a second target in retinoblastoma cells in our in vitro testing pipeline. GD2 is known to be expressed on neuroectodermal tumors [22] and is, besides IL13RA2 and mesothelin, one of the few CAR-T cell targets to have produced solid tumor regression. [42–44] Since our sample cohort used to assess CD171 was long-term stored, formalin-fixed and paraffin embedded material it was inappropriate for GD2 immunohistochemistry. However, it has previously been shown that retinoblastoma derived metastases in patients express high GD2 levels even at the time of minimal residual disease after retinoblastoma therapy is completed. [19, 20] We think it is highly unlikely that GD2 is not frequently expressed in retinoblastoma, and therefore, it is a suitable target for CAR-T cell therapy. Further, flow cytometry analysis of our retinoblastoma cell line panel revealed that all cell lines expressed GD2 (Fig. 4a). More than 90% of cells expressed GD2 in 6 of the 11 cell lines, including WERI-Rb1 and RBL15 (96.0–99.9%), while approximately half the cells expressed GD2 in 4 of 11 cell lines, including RB355 (mean = 52.9%, range = 41–65%). Investigating the number of GD2 antigen-binding sites per cell showed that WERI-Rb1 and RBL15 cells provided > 80,000 target molecules per cell, with respective ranges of 136,412 – 146,692.0 and 62,770–100,846 targets/cell (Fig. 4b). Consistent with the lower GD2 expression measured in the RB355 cell line, only 3778–4775 target molecules per cell were detected. Even with the lower GD2 expression in some retinoblastoma cell lines, these data support GD2 as potential second target for retinoblastoma-specific CAR-T cell therapy.

**Fig. 5:**
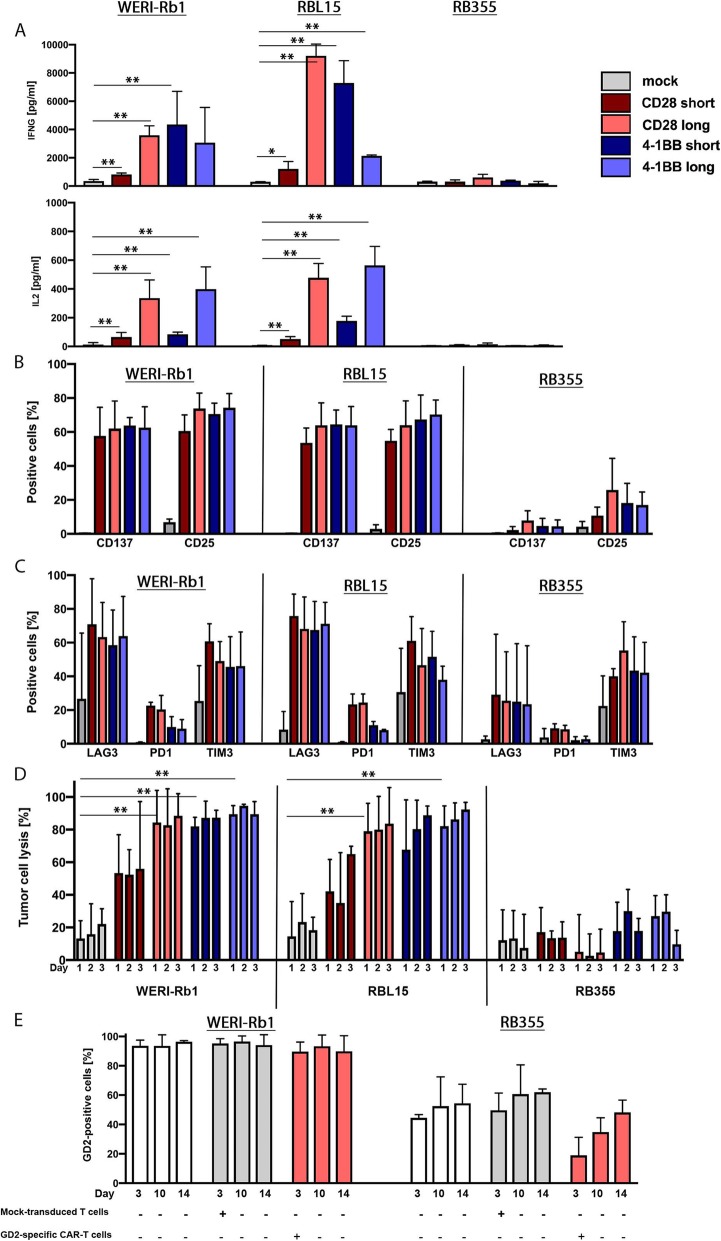
GD2-specific CAR-T cells kill retinoblastoma cells in vitro without causing antigen loss in WERI-Rb1. **a** IFNG and IL2 release of CAR-T cells following a 24-h co-culture at a 2:1 E:T ratio with retinoblastoma cell lines (mean ± SD, n = 3). Cell surface expression of activation markers CD137 and CD25 (**b**) and inhibitory receptors LAG3, PD1 and TIM3 (**c**) on CAR-T cells is shown upon a 24-h co-culture at a 2:1 E:T ratio (mean ± SD, n = 3). Color-coding is the same as used in **a**. **d** Cytotoxicity of GD2-specific CAR-T cells is displayed in comparison to mock-transduced T cells. Cytotoxicity was measured by a luciferase-based killing assay following a 24, 48 and 72 h co-culture at a 2:1 E:T ratio (mean ± SD, n = 3). Color-coding is the same as used in **a**. **e** Cell line-dependent loss of GD2 cell surface expression is shown after a 72-h co-culture as well as day 10 and 14 after CAR-T cell removal (n = 3). *, p ≤ 0.5; **, p ≤ 0.01

To investigate the potential efficacy of GD2-specific CAR-T cells against retinoblastoma cells, we generated CAR-T cells targeting the 14.2GA-GD2 epitope with the same four different CAR constructs used in the CD171-specific CAR-T cells (see Additional file 4: Figure S3B). We again used the WERI-Rb1, RBL15 and RB355 cell lines in co-culture with GD2-specific CAR-T cells before investigating T cell activation and tumor cell killing efficacy in vitro. Similar to CD171-specific CAR-T cells, encounter with WERI-Rb1 and RBL15 induced IFNG and IL2 release from GD2-specific CAR-T cells but not mock-transduced T cells used as negative controls (Fig. 5a). Co-culture with the GD2-negative NALM-6 cell line served as control for antigen-specificity of GD2-specific CAR-T cells (Additional file 5: Figure S4C + D). In concordance with the low antigen levels detected by flow cytometry analysis, encounter with RB355 induced neither IFNG nor IL2 release from GD2-specific CAR-T cells. Activation markers (CD25 and CD137) on T cells harboring the GD2-specific CAR constructs were also assessed by flow cytometry analysis after retinoblastoma encounter. Any of the 3 retinoblastoma cell lines tested induced both activation markers in co-cultured GD2-specific CAR-T cells (Fig. 5b). The WERI-Rb1 and RBL15 cell lines were more capable of activating CAR-T cells than the RB355 cell line, in line with its lower target expression. Expression of activation markers on GD2-specific CAR-T cells was stronger increased upon encounter with WERI-Rb15 and RBL15 cells than on CD171-specific CAR-T cells. Co-culture with WERI-Rb1 induced a 3-fold higher CD25 (range = 2.3-fold-4.8-fold) and 1.9-fold higher CD137 (range = 1.5-fold-3.1-fold) expression on GD2-specific CAR-T cells in comparison to CD171-specific CAR-T cells, while co-culture with RBL15 cells induced a 1.3-fold higher (range = equal expression-1.8-fold) and 1.3-fold higher CD137 (range = equal-expression-1.8-fold) expression of activation markers. Expression of the LAG3, TIM3 and PD1 inhibitory receptors were also assessed after retinoblastoma encounter by flow cytometry analysis. Co-culture with any of the 3 retinoblastoma cell lines more strongly induced expression of all 3 inhibitory receptors in most GD2-specific CAR-T cells than was observed in CD171-specific CAR-T cells (Fig. 5c). Encounter with WERI-Rb1 induced 3.6-fold higher LAG3 expression in GD2-specific compared to CD171-specific CAR-T cells, whereas RBL15 encounter induced 1.6-fold higher LAG3 expression. Interestingly, within GD2-specific CAR-T cells, PD1 expression on CAR-T cells harboring the CD28 co-stimulatory domain was 2.2-fold (WERI-Rb1), 2.5-fold (RBL15) and 1.5-fold (RB355) higher compared to CAR-T cells with 4-1BB co-stimulation domain. Our results demonstrate that exposure to retinoblastoma cells activated GD2-specific CAR-T cells and induced cytokine release, dependent on antigen density.

To test the ability of GD2-specific CAR-T cells to kill retinoblastoma cells in vitro, we assessed cytotoxic efficacy using the luciferase-based reporter assay. GD2-specific CAR-T cells were co-cultured in a 2:1 effector:target ratio with retinoblastoma reporter cells before assessing the biophotonic signal released by the remaining viable tumor cells after 24 h, 48 h and 72 h. GD2-specific CAR-T cells killed significantly more WERI-Rb1 and RBL15 cells than the mock-transduced negative control (Fig. 5d). Cytotoxicity against RB355 cells was weaker, in line with the lower target expression. The GD2-negative NALM-6 cell line was not killed by GD2-specific CAR-T cells (Additional file 5: Figure S4D). Dose-dependent cytotoxicity of GD2-specific CAR-T cells was demonstrated in co-culture with RBL15 cells in different effector:target ratios. Cytotoxicity ranged from 90.1% in a 5:1 effector:target ratio to 66% (1:1 effector:target ratio) to 1.5% (1:10 effector:target ratio; Additional file 6: Figure S5). These experiments demonstrate that CAR-T cells targeting GD2 efficiently kill retinoblastoma cells in vitro in an antigen density-dependent manner.

The impact of different spacer and co-stimulatory domains on cytokine release, activation, inhibitory receptor expression and killing ability of T cells targeting GD2 was also assessed. Similar to CD171-specific CAR-T cells, T cells harboring the long spacer and CD28 co-stimulatory domain released more IFNG and IL2 after encounters of any tested cell line than T cells harboring the short spacer and CD28 co-stimulatory domain (Fig. 5a), indicating superior effector functionality of T cells equipped with the GD2-long-CD28 construct. T cells harboring the long spacer with either co-stimulatory domain released comparable amounts of IL2 after encounters with WERI-Rb1 and RBL15. T cells harboring all constructs except the GD2-short-CD28 CAR construct were comparably well activated by WERI-Rb1 or RBL15 cell co-culture (Fig. 5b). Inhibitory receptor expression was comparably induced in T cells harboring all constructs, except that PD1 expression was stronger in T cells harboring the CD28 co-stimulatory domain (Fig. 5c). The CAR construct used did not have as large an impact on the CAR-T cell ability to kill retinoblastoma cells as it had on cytokine release by the T cells. Most GD2-specific CAR-T cells were able to kill nearly 90% (Fig. 5d) of the retinoblastoma cells underlining the potency of GD2-specific CAR-T cell therapy against retinoblastoma. Although the CAR construct had less impact on efficacy of GD2-specifc compared with CD171-specific CAR-T cells, the GD2-long-CD28 construct performed best in vitro and was selected for the remaining experiments.

Having verified cytokine release, activation and cytolytic capacity we also assessed tumor escape mechanisms of retinoblastoma cell lines following GD2-specific CAR-T cell encounter. As done before for the CD171-specific CAR-T cells, we investigated GD2 expression on WERI-Rb1 and RB355 cells via FACS analysis at several time points after transient co-culture with GD2-specific CAR-T cells (Fig. 5e, Additional file 8: Figure S7). Interestingly, while transient encounter with GD2-specific CAR-T cells downregulated the already low GD2 expression on RB355 cells by more than half when assessed on day 3 concomitantly with CAR-T cell removal, WERI-Rb1 cell lines did not change GD2 expression. RB355 cells regained GD2 expression by day 14, with antigen levels comparable to untreated cells. GD2 antigen expression may only be differentially regulated in cells with low antigen densities, reducing the risk of potential immune evasion when GD2 is targeted in retinoblastoma cells.

### Sequential CAR-T cell treatment counteracts antigen loss and improves cytotoxicity against retinoblastoma cells

**Fig. 6:**
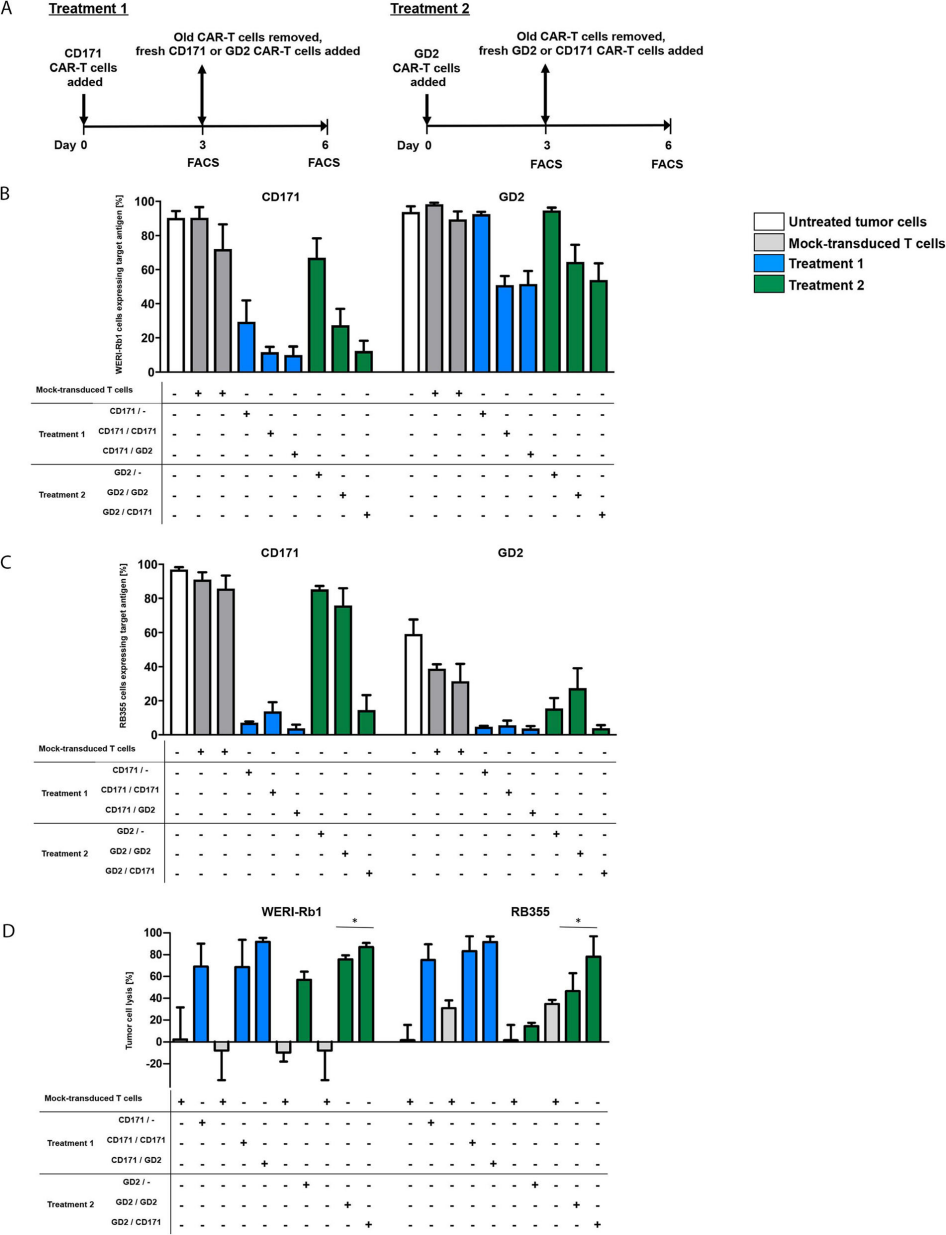
Sequential targeting of different antigens increases CAR-T cell efficacy in vitro. **a** Scheme of the experimental setting depicting treatment option I and II. **b** CD171 and GD2 cell surface expression on WERI-Rb1 cell line is shown on day 3 and 6 after treatment. Retinoblastoma cells were initially treated on day 0 and sequential treatment was conducted on day 3 at a 1:5 E:T ratio, respectively (mean ± SD, *n* = 3). **C.** For cell line RB355, CD171 and GD2 expression is illustrated as in **b** (*n* = 3). **d** Lysis of WERI-RB1 and RB335 is shown after initial treatment with CD171- or GD2-specific CAR-T cells and on day 3 and after sequential treatment on day 6 (1:5 E:T ratio, *n* = 3). Mock-transduced T cells serve as negative control. Treatment is specified in the table below. *, *p* ≤ 0.5; **, *p* ≤ 0.01

We investigated whether sequentially treating retinoblastoma cells with CAR-T cells targeting the same or different target antigens could reduce escape via CD171 or GD2 downregulation on retinoblastoma cells. All 11 retinoblastoma cell lines showed CD171^+^GD2^+^-positive cells as analyzed by flow cytometry (range = 11–96%, Additional file 9: Figure S8). T cells harboring CD171- or GD2-specific constructs with the long spacer and CD28 co-stimulatory domains were used, since these performed best in single-treatment experiments. CAR-T cells targeting either CD171 (treatment 1) or GD2 (treatment 2) were co-cultured with retinoblastoma cells in a 1:5 effector:target ratio for 3d before examining CD171 and GD2 expression on tumor cells (Fig. 6a). CAR-T cells were removed on day 3 and replaced with CAR-T cells targeting either the same or the alternate antigen. CD171 and GD2 expression was measured again on day 6. Sequential treatment initiated with CD171-specific CAR-T cells (treatment 1) reduced CD171 expression on WERI-Rb1 cells to 29.3% on day 3, and further reduced CD171 expression to only < 12% of cells on day 6 regardless of whether CAR-T cells targeting CD171 or GD2 were applied on day 3 (Fig. 6b). Initiating sequential treatment with GD2-specific CAR-T cells (treatment 2) did not downregulate CD171 expression on WERI-Rb1 cells on day 3, but reduced WERI-Rb1 cells expressing CD171 to 27.4% on day 6 if GD2-specific CAR-T cells were re-applied and to 12.5% if CD171-specific CAR-T cells were applied at day 3. GD2 expression on WERI-Rb1 cells remained unaffected on day 3, regardless of the antigen being targeted (treatment 1 or 2), but treatment on day 3 with either CD171- or GD2-specific CAR-T cells downregulated GD2 expression on WERI-Rb1 cells to similar levels. Exposing RB355 cells to CD171-specific CAR-T cells (treatment 1) reduced CD171 and GD2 expression to < 8% of the cells by day 3 (Fig. 6c). The very low levels of target expression at day 3, made accurate assessment of further target reduction on day 6 difficult. Interestingly, GD2 expression levels on RB355 cells were more strongly reduced on day 3 by CAR-T cells targeting CD171 than GD2, indicating reduction of antigen expression was independent of the CAR target. Sequentially applied GD2-specific CAR-T cells (treatment 2) barely influenced CD171 expression on RB355 cells on days 3 or 6, and only strongly reduced expression of either target if treated with CD171-specific CAR-T cells on day 3. Our data demonstrate that antigen downregulation is triggered by CAR-T cell exposure, but that downregulation does not always depend on the antigen targeted by the CAR.

In parallel, we assessed cytotoxic efficacy of sequential CAR-T cell treatment targeting the same or 2 different antigens using the same luciferase-based reporter assay for tumor cell killing as in our monotreatment experiments. The same treatment regimens (Fig. 6a) were conducted with 1:5 effector:target ratios for CAR-T cells and WERI-Rb1 or RB355 cells (Fig. 6d). CD171- and GD2-specific CAR-T cells showed comparable killing efficacy of WERI-Rb1 cells during treatment 1 and 2. Switching from GD2- to CD171-specific CAR-T cells increased tumor cell lyses by 30% from day 3 to day 6 while a sequential treatment with GD2-specfifc CAR-T cells showed an increase of tumor lysis of only 19% indicating a benefit for administration of sequential CAR-T cell therapy targeting different antigens. Analyzing CD171- and GD2-specific CAR-T cell killing ability of RB355 cells revealed higher cytotoxicity of CD171- than of GD2-specific CAR-T cells during treatment 1 and 2. Treatment with GD2-specific CAR-T cells followed by subsequent CD171-specific CAR-T cell treatment displayed a significantly higher tumor lysis compared to a second GD2-specific CAR-T cell treatment. Our in vitro analyses support that sequentially targeting multiple antigens with CAR-T cells increases treatment efficacy, with the greatest benefit produced by targeting CD171 before GD2, even in cells with initial low-density GD2 expression.

## Discussion

We present preclinical evidence that CAR-T cell therapy targeting retinoblastoma-specific antigens could be a promising option to treat both primary tumors as well as metastasized or metastasizing malignant cells. CD171- and GD2-specific CAR-T cells were strongly activated by retinoblastoma cell encounter and demonstrated potent killing efficacy of retinoblastoma cells in vitro dependent on target antigen expression*.* Though all tested CAR constructs demonstrated equal activation and killing ability for either target, the CAR construct harboring the long extracellular spacer in combination with the CD28 co-stimulatory domain, tended to release higher amounts of effector cytokines, although this difference did not reach statistical significance While CD171 expression was completely lost on all cell lines following CAR-T cell treatment, GD2 was only down-regulated in cell lines initially harboring low GD2 levels. Here, we reveal enhanced retinoblastoma cell killing in alternating the CAR-T cell killing in sequential CAR-T cell challenges.

Watanabe et al. previously demonstrated for CD20-specific CAR-T cells that antigen density on acute lymphatic leukemia cell lines influenced CAR-T cell efficacy. [45] The same was shown by Fry et al. in patients with CD22-diminished or -negative leukemia blasts, who did not respond to CD22-specific CAR-T cell therapy. [46] Turrati et al. developed a cellular model where HER2 antigen expression could be increased, which caused enhanced CAR-T cell activity. [47] Here, we also show antigen expression level was correlated with cytotoxicity for GD2-specific CAR-T cells, since low GD2-expressing RB355 cells were only marginally killed, while WERI-Rb1 cells with higher GD2 expression elicited efficient killing.

The non-signaling extracellular spacer plays a pivotal role in tumor recognition: as each new scFv and target molecule on tumor membrane define a distinct biophysical synapse, a unique adjustment is needed for every target antigen. Hudecek et al. could previously demonstrate that T cells harboring a short spacer-containing CD19-CAR were able to eradicate tumor cells in vivo while the same dose of CD19-specific CAR-T cells with a long spacer failed to do so. [15] We previously showed that levels of activation and cytotoxicity for CD171-specific CARs varied depending on the extracellular spacer length when targeting neuroblastoma. [36] In this previous study, we compared CD171-specific CAR constructs that all harbored the 4-1BB co-stimulatory domain but different spacer length. The CAR construct with the long spacer domain performed best in in vitro assays, but, interestingly, the superiority of the long spacer CAR was reversed in vivo, most likely due to activation-induced cell death caused by recursive antigen exposure. The data we present here showed no clear superiority for any of the extracellular spacer length, irrespectively of target cell line or amount of antigen expression. However, investigation of the different CAR constructs in preclinical mouse models might reveal differences, since CAR-T cell performance has been shown to be influenced by additional factors such as CAR-T cell expansion and persistence in vivo. [48] The mechanisms by which different co-stimulatory domains influence T cell expansion, function and persistence are not yet fully understood, but the CD28 co-stimulatory domain is associated with enhanced anti-tumor efficacy and T cell functionality and enhanced T cell survival and persistence is attributed to the 4-1BB co-stimulatory domain. [49] Hudecek et al. revealed higher cytokine release for ROR1-specific CAR-T cells harboring a CD28 co-stimulatory domain compared to T cells with a 4-1BB co-stimulatory domain. [50] Long et al. previously showed that tonic signaling in CAR-T cells harboring a CD28 co-stimulatory domain led to T cell exhaustion and reduced functionality in vivo, which could be ameliorated by incorporation of 4-1BB into GD2-CAR-T cells. [40] In line with these findings, our data depicts increased PD1 expression on GD2-specific CAR-T cells harboring the CD28 co-stimulatory. However, from our functional analyses of GD2- and CD171-specific CAR-T cells, we conclude that all tested CD171- and GD2-specific CAR constructs, irrespective of the co-stimulatory domain, against retinoblastoma in vitro*.* Further testing in preclinical mouse models will reveal whether there is a ranking between CD171- and GD2-specific CARs equipped with 4-1BB or CD28 costimulatory domains against retinoblastoma.

Differences in CAR-T cell exhaustion of CAR-T cells with different scFvs were previously demonstrated by Yin et al., who analyzed EGFRvIII- and IL-13Rα2-targeting CAR-T cells against the same tumor model. [51] By using different scFvs, Yin et al. showed a higher expression of the immune checkpoint molecule CTLA-4 in higher stimulated CAR-T cells, while TIM-3 and PD1 are predominantly expressed on less activated CAR-T cells. [51] In our study, the differences between the expression of inhibitory receptors could also be explained by the different scFvs used. Further, the activation level is not significantly higher but a trend towards higher cytokine production and expression of activation markers can be observed after 24 h of co-culture and may reach significant difference after 48 h.

On the way to translate these findings into the clinic, in vivo experiments need to further explore potential toxicities, e.g. cytokine release syndrome, in context of an ocular tumor.

In general, tumor cells are capable of evading targeted immune therapy by downregulating the targeted antigen leading to reduced tumor recognition and rendering CAR-T cell therapy inefficient. [42, 52] Clinical application of sequential lymphoma-specific CAR-T cells was recently described by Shalabi et al. [53] Targeting B-cell malignancies with CD19-specific CAR-T cells resulted in outgrowth of antigen-negative variants. However, subsequent targeting with a second CAR-specificity could achieve cancer regression and prolonged survival of cancer patients, highlighting the importance of sequential tissue evaluation through the course of treatment. Fry et al. revealed CD22-specific CAR-T cell therapy is an option for patients relapsing after CD19-directed treatment. [46] Brown et al. demonstrated a response in a patient with recurrent multifocal glioblastoma using sequentially administered CAR-T cells having the same target specificity. [44] While loss of both antigens was observed in RB355 cell lines, the WERI-Rb1 cell line did not downregulate GD2 levels independently of CAR specificity. The different expression pattern could be related to differences in GD2 expression between the cell lines. As RB355 cells generally express low levels of GD2 and RB355 cells express fewer antigen molecules per cell, the downregulation may be detected earlier than in the high GD2 expression background on WERI-Rb1 cells. The subsequent GD2 upregulation after 10 days may be due to an indispensable GD2 function in the cell population. GD2 is associated with tumorigenesis as well as cancer cell proliferation and invasion. [24] Here we demonstrate that sequentially administering CAR-T cells targeting different antigens achieves higher CAR-T cell killing ability.

## Conclusions

Our data revealed CD171 and GD2 as effective targets for CAR-T cell therapy for retinoblastoma in vitro. We demonstrated the risk potential of antigen loss on targeted tumor cells after CAR-T cell treatment, but also show that sequentially switching antigen specificity in the CAR-T cell therapy provides a striking benefit for retinoblastoma cell killing ability. This work provides the basis for in vivo testing to select the most beneficial regimens and target combinations on the way to establishing CAR-T cell therapy for retinoblastoma.

## Supplementary information

**Additional file 1: Figure S1.** Immunohistochemical CD171-staining of human kidney tissue serves as positive control. (JPG 138 kb)

**Additional file 2:** Supplementary Method Description. Detailed description of immunohistochemically methods including detection of CD171- and CD3-positive cells and quantification of CD3^+^ cells in primary retinoblastoma tissue sections. (DOCX 20 kb)

**Additional file 3: Figure S2.** Classification of CD171-positive primary retinoblastoma tissue sections in various frequencies. Samples were categorized in < 5%, 5–50%, 51–90 and > 90% CD171-positive samples. (JPG 100 kb)

**Additional file 4: Figure S3.** CAR-transduction efficacy after enrichment. CD8^+^ bulk cells were lentivirally transduced with CD171- (**A**) and GD2-specific (**B**) CAR-constructs, respectively. After enrichment, detection of CAR positive CD8^+^ cells was performed with fluorochrome-conjugated cetuximab antibody. Untransduced T cells serve as negative control (labeled as mock). (JPG 261 kb)

**Additional file 5: Figure S4.** B-cell lymphoma cell line NALM-6 serves as negative control**. A.** NALM-6 cells do not express antigens CD171 and GD2 as analyzed by flow cytometry**.** IFNG and IL2 release of CD171- **(B)** and GD2-specific CAR-T cells **(C)** following a 24-h co-culture at a 2:1 E:T ratio with NALM-6 cells compared to RBL15 retinoblastoma cells (mean ± SD, *n* = 1 in technical triplicates). **D.** Cytotoxicity of CD171-and GD2-specific CAR-T cells is displayed in comparison to mock-transduced T cells. Cytotoxicity was measured by a luciferase-based killing assay following a 24 h co-culture at a 2:1 E:T ratio compared to RBL15 cells (mean ± SD, n = 1 in technical triplicates). Color-coding is the same as used in B. (JPG 128 kb)

**Additional file 6: Figure S5.** CD171- and GD2-specific CAR-T cells show dose-dependent cytotoxicity. Tumor lysis of CD171- and GD2-specific CAR-T cells (CD28 long-spacer) as well as simultaneous treatment with both constructs in comparison to mock-transduced CAR-T cells after co-culture with RBL15. Tumor lysis was measured by a luciferase-based killing assay following a 24 h co-culture at different effector:target ratios (mean ± SD, n = 1 in technical triplicates). (JPG 44 kb)

**Additional file 7: Figure S6.** CD171-specific CAR-T cell treatment leads to reduction of CD171-positive cells. CD171 expression on WERI-Rb1 and RB355 cells on day 3 of co-culture with CD171- and GD2-specific CAR-T cells compared to untreated cells and treatment with untransduced T cells (mock) as analyzed by flow-cytometry. (JPG 88 kb)

**Additional file 8: Figure S7.** GD2-specific CAR-T cell treatment effects CD171 and GD2 expression on RB355 cells while antigen expression on WERI-Rb1 cells remains unchanged. GD2 expression in WERI-Rb1 and RB355 cells on day 3 of co-culture with CD171- and GD2-specific CAR-T cells compared to untreated cells and treatment with untransduced T cells (mock) as analyzed by flow-cytometry. (JPG 96 kb)

**Additional file 9: Figure S8.** CD171 and GD2 expression profiles on retinoblastoma cell lines. FACS-based data of CD171 and GD2 expression for all analyzed retinoblastoma cell lines. (JPG 79 kb)

## Data Availability

Data supporting the results reported in the article are available from Annette Künkele on reasonable request.

## Abbreviations

CAR: Chimeric antigen receptor
CD: Cluster of differentiation
EBV: Epstein-barr virus
EGFRt: Truncated epidermal growth factor receptor
ELISA: Enzyme-linked immunosorbent assay
FACS: Fluorescence-activated cell sorting
FCS: Fetal calf serum
ffLuc: Firefly luciferase
GFP: Green fluorescent protein
ICRB: International classification of retinoblastoma
IFNG: Interferon-γ
IHC: Immunohistochemistry
LAG3: Lymphocyte-activation gene 3
mAb: Monoclonal antibody
OKT3: Muromonab-CD3
PE: Phycoerythrin
RLU: Relative light unit
scFv: Single chain variable fragment
SIN: Self-inactivating
T2A: Trichocyst matrix protein T2A
TIM3: T-cell immunoglobulin and mucin-domain containing-3
TMLCL: EBV-transformed lymphoblastoid cell line
