# Supplementary figures and images for: CD171- and GD2-specific CAR-T cells potently target retinoblastoma cells in preclinical in vitro testing
Source: BMC Cancer. 2019 Sep 9;19:895. doi: 10.1186/s12885-019-6131-1 (PMC6732842; doi:10.1186/s12885-019-6131-1)

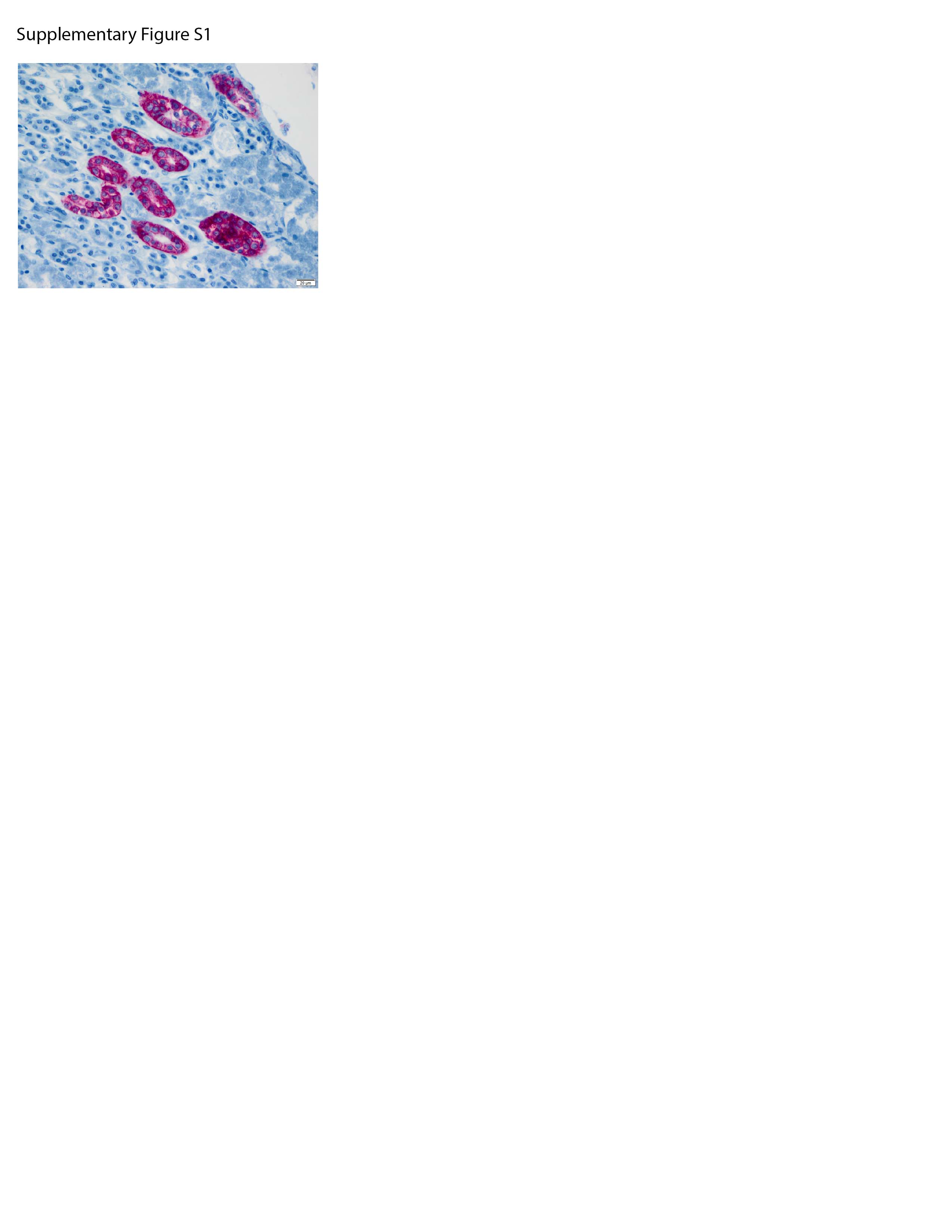

Supplement: Supplementary file 1 — Additional file 1: Figure S1. Immunohistochemical CD171-staining of human kidney tissue serves as positive control. (JPG 138 kb) [file 12885_2019_6131_MOESM1_ESM.jpg]

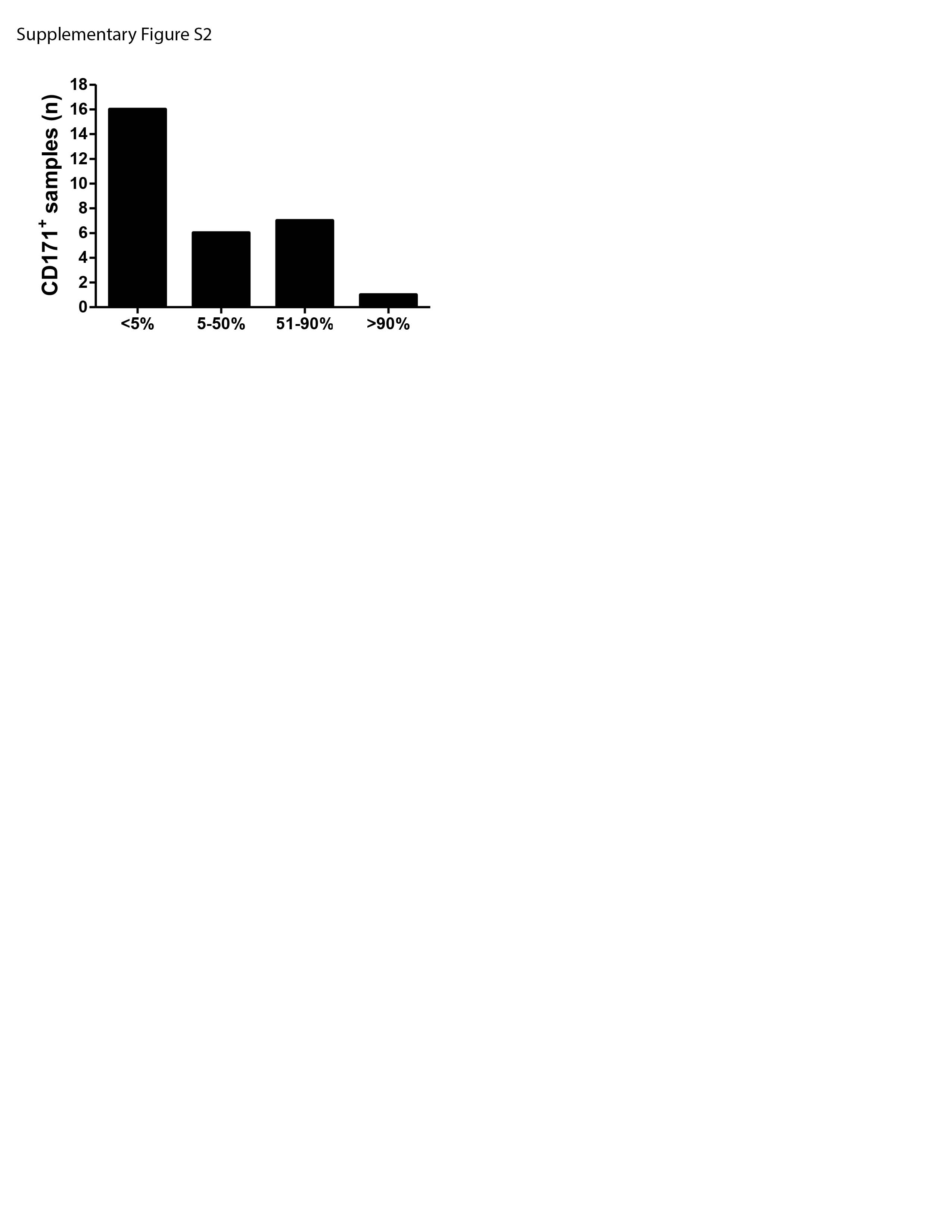

Supplement: Supplementary file 3 — Additional file 3: Figure S2. Classification of CD171-positive primary retinoblastoma tissue sections in various frequencies. Samples were categorized in < 5%, 5–50%, 51–90 and > 90% CD171-positive samples. (JPG 100 kb) [file 12885_2019_6131_MOESM3_ESM.jpg]

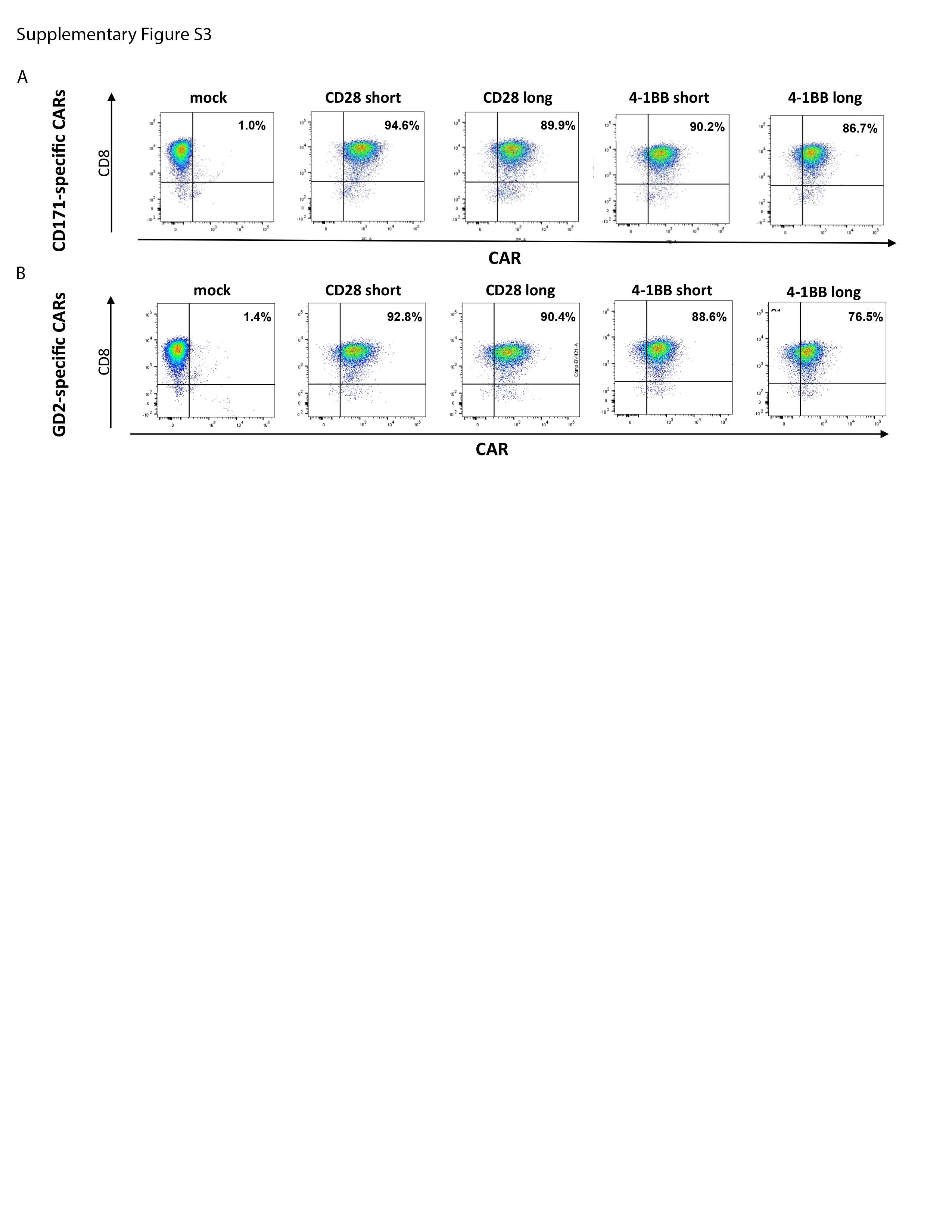

Supplement: Supplementary file 4 — Additional file 4: Figure S3. CAR-transduction efficacy after enrichment. CD8+ bulk cells were lentivirally transduced with CD171- (A) and GD2-specific (B) CAR-constructs, respectively. After enrichment, detection of CAR positive CD8+ cells was performed with fluorochrome-conjugated cetuximab antibody. Untransduced T cells serve as negative control (labeled as mock). (JPG 261 kb) [file 12885_2019_6131_MOESM4_ESM.jpg]

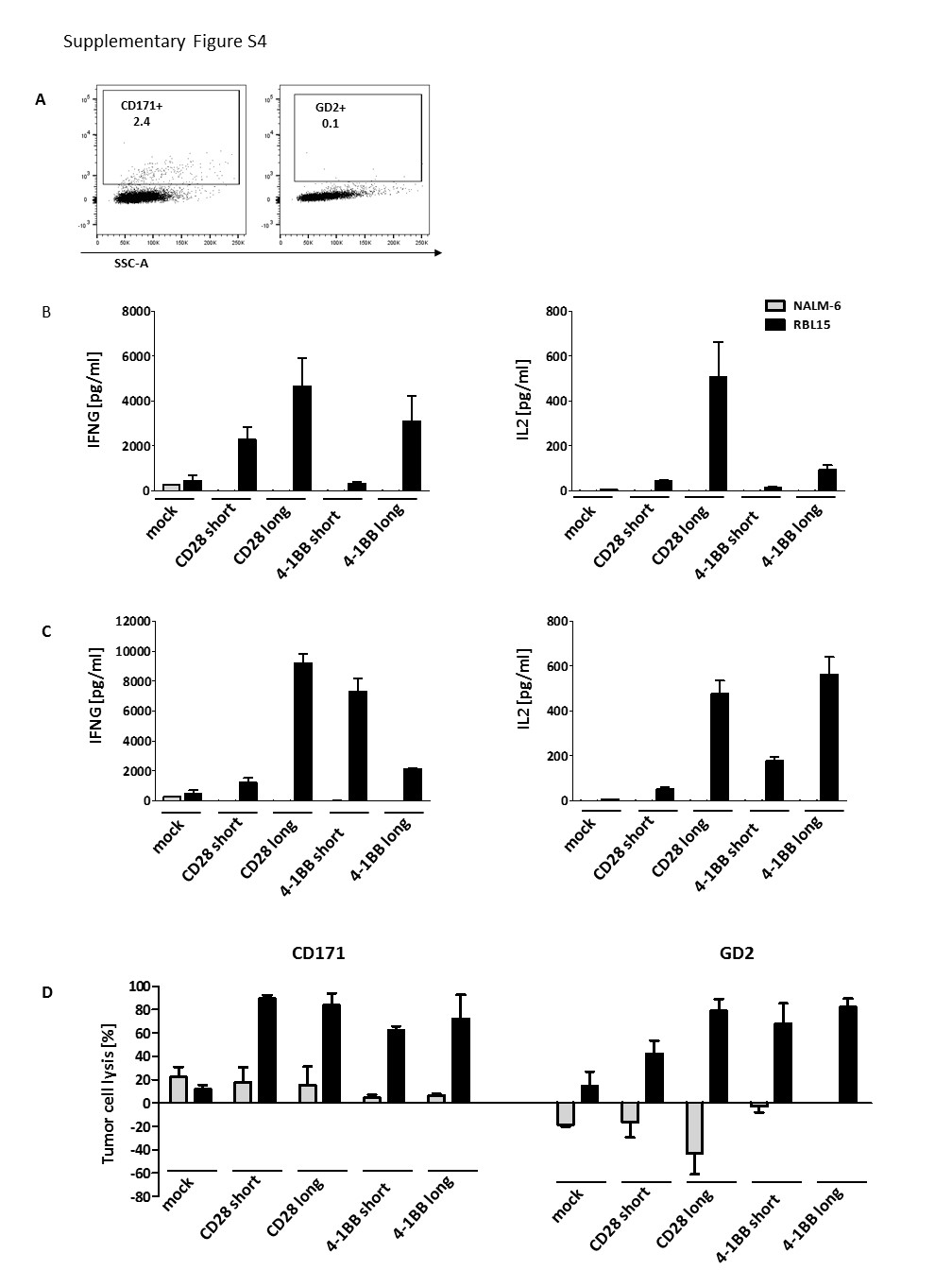

Supplement: Supplementary file 5 — Additional file 5: Figure S4. B-cell lymphoma cell line NALM-6 serves as negative control. A. NALM-6 cells do not express antigens CD171 and GD2 as analyzed by flow cytometry. IFNG and IL2 release of CD171- (B) and GD2-specific CAR-T cells (C) following a 24-h co-culture at a 2:1 E:T ratio with NALM-6 cells compared to RBL15 retinoblastoma cells (mean ± SD, n = 1 in technical triplicates). D. Cytotoxicity of CD171-and GD2-specific CAR-T cells is displayed in comparison to mock-transduced T cells. Cytotoxicity was measured by a luciferase-based killing assay following a 24 h co-culture at a 2:1 E:T ratio compared to RBL15 cells (mean ± SD, n = 1 in technical triplicates). Color-coding is the same as used in B. (JPG 128 kb) [file 12885_2019_6131_MOESM5_ESM.jpg]

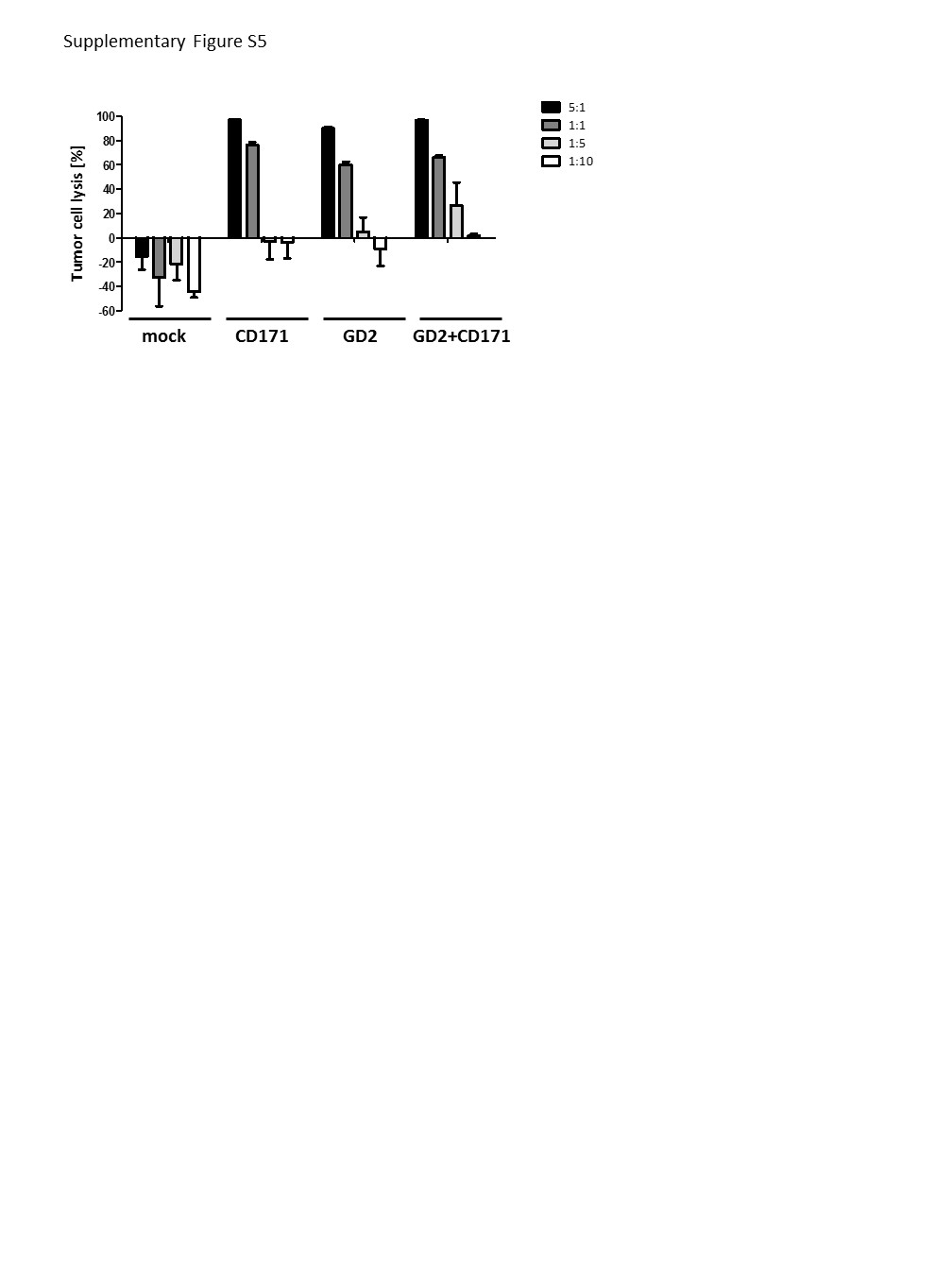

Supplement: Supplementary file 6 — Additional file 6: Figure S5. CD171- and GD2-specific CAR-T cells show dose-dependent cytotoxicity. Tumor lysis of CD171- and GD2-specific CAR-T cells (CD28 long-spacer) as well as simultaneous treatment with both constructs in comparison to mock-transduced CAR-T cells after co-culture with RBL15. Tumor lysis was measured by a luciferase-based killing assay following a 24 h co-culture at different effector:target ratios (mean ± SD, n = 1 in technical triplicates). (JPG 44 kb) [file 12885_2019_6131_MOESM6_ESM.jpg]

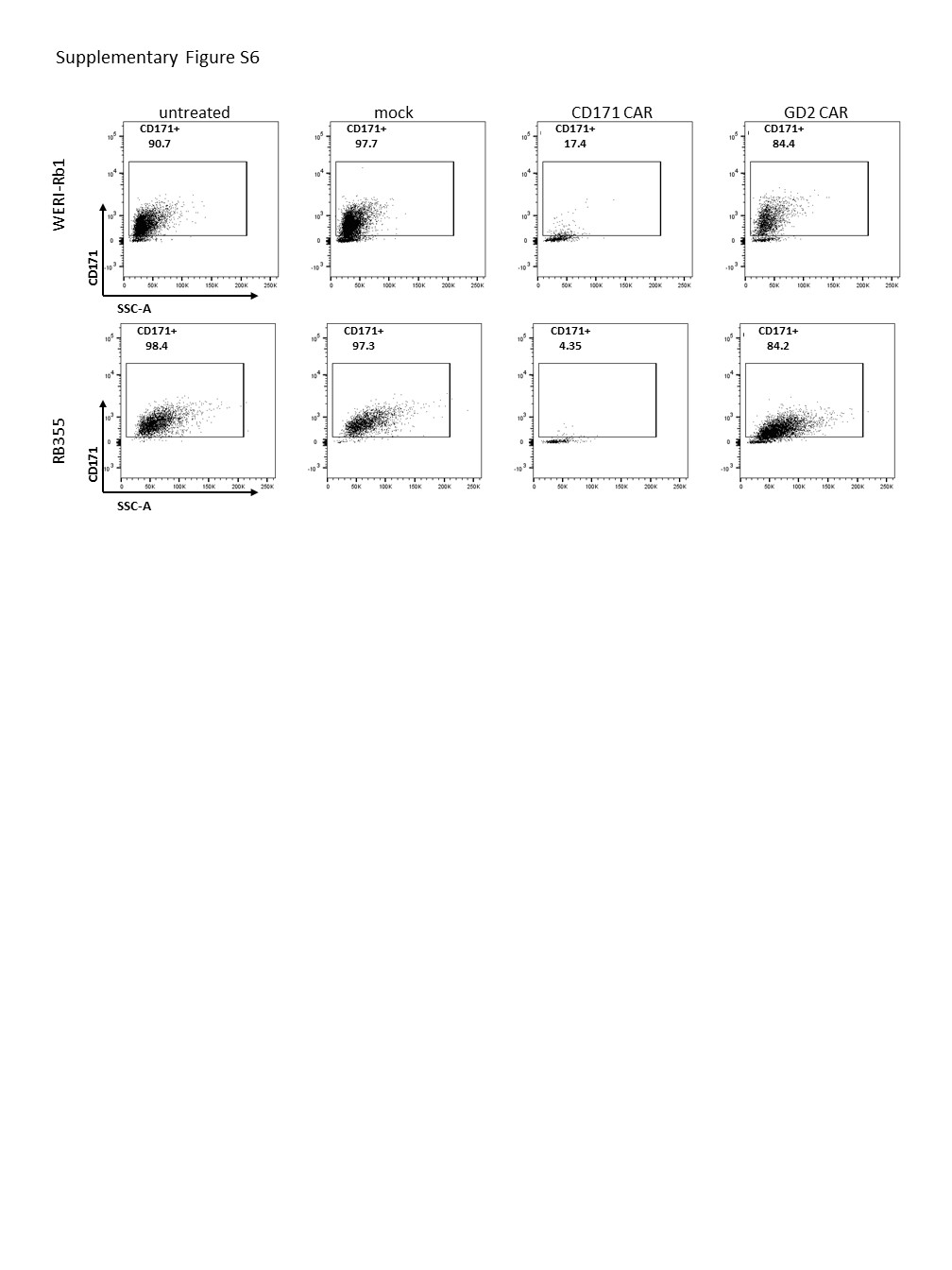

Supplement: Supplementary file 7 — Additional file 7: Figure S6. CD171-specific CAR-T cell treatment leads to reduction of CD171-positive cells. CD171 expression on WERI-Rb1 and RB355 cells on day 3 of co-culture with CD171- and GD2-specific CAR-T cells compared to untreated cells and treatment with untransduced T cells (mock) as analyzed by flow-cytometry. (JPG 88 kb) [file 12885_2019_6131_MOESM7_ESM.jpg]

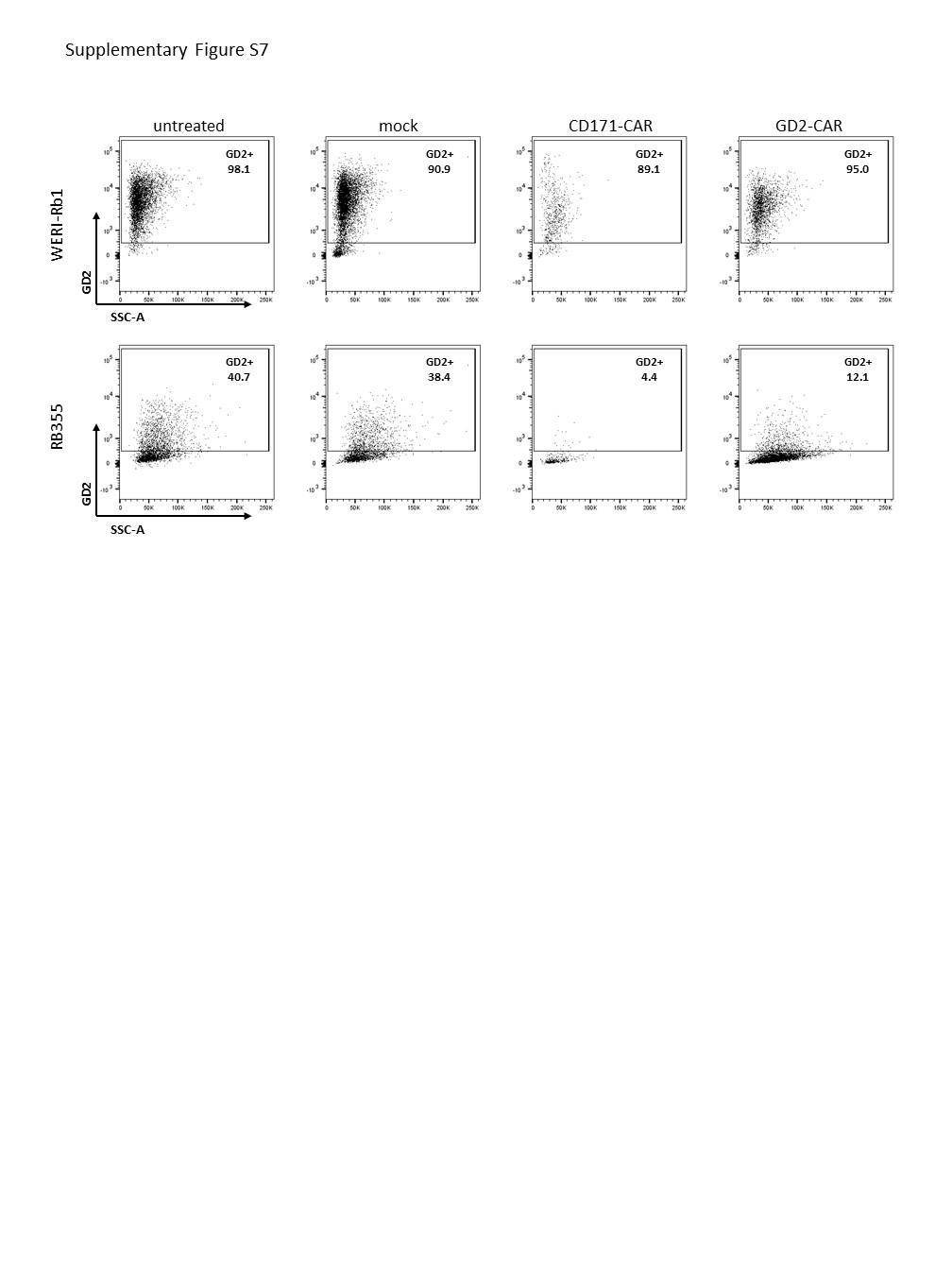

Supplement: Supplementary file 8 — Additional file 8: Figure S7. GD2-specific CAR-T cell treatment effects CD171 and GD2 expression on RB355 cells while antigen expression on WERI-Rb1 cells remains unchanged. GD2 expression in WERI-Rb1 and RB355 cells on day 3 of co-culture with CD171- and GD2-specific CAR-T cells compared to untreated cells and treatment with untransduced T cells (mock) as analyzed by flow-cytometry. (JPG 96 kb) [file 12885_2019_6131_MOESM8_ESM.jpg]

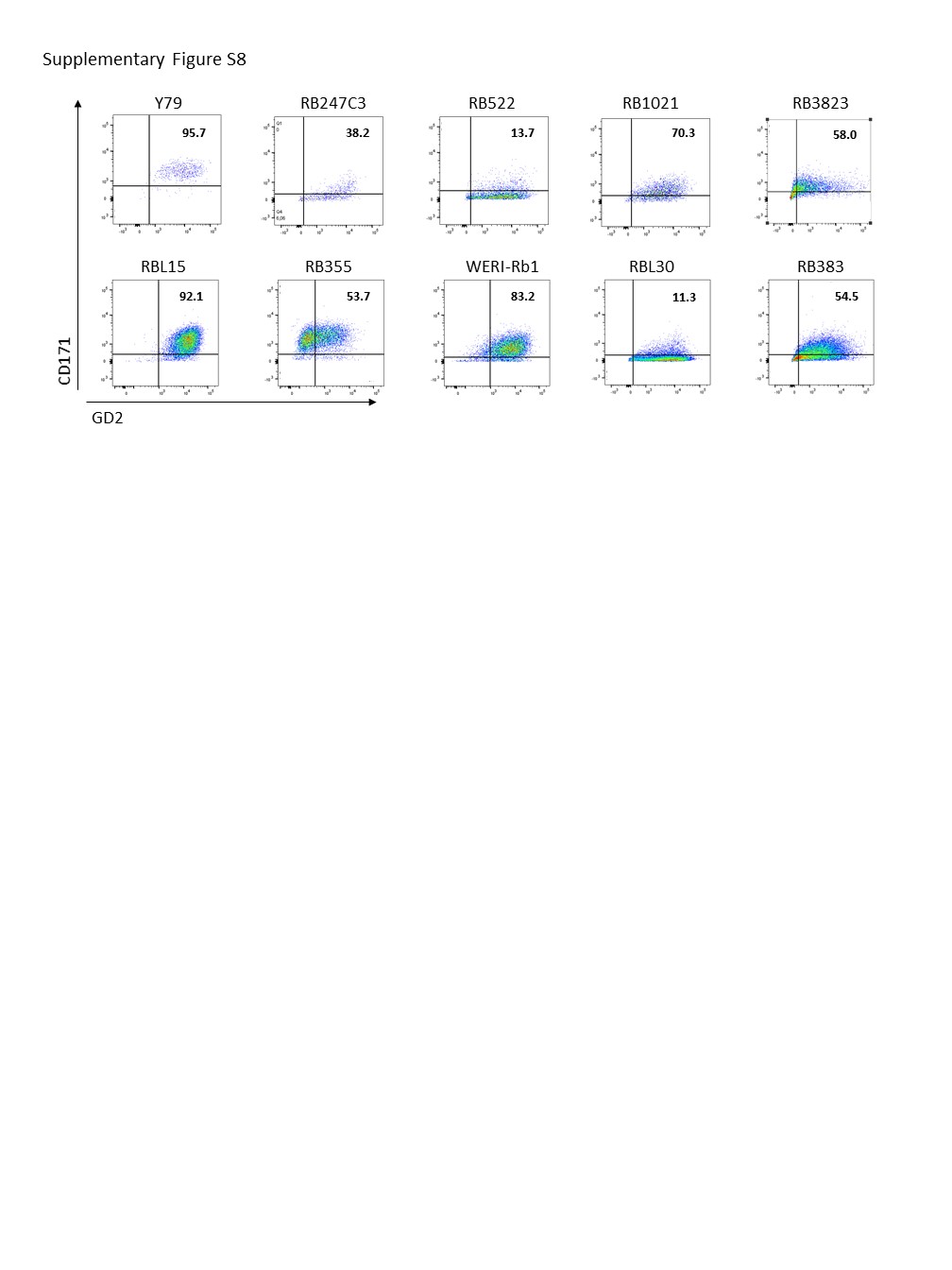

Supplement: Supplementary file 9 — Additional file 9: Figure S8. CD171 and GD2 expression profiles on retinoblastoma cell lines. FACS-based data of CD171 and GD2 expression for all analyzed retinoblastoma cell lines. (JPG 79 kb) [file 12885_2019_6131_MOESM9_ESM.jpg]
