# Supplementary material for: CD171- and GD2-specific CAR-T cells potently target retinoblastoma cells in preclinical in vitro testing
Source: BMC Cancer. 2019 Sep 9;19:895. doi: 10.1186/s12885-019-6131-1 (PMC6732842; doi:10.1186/s12885-019-6131-1)
Supplement: Supplementary file 2 — Additional file 2: Supplementary Method Description. Detailed description of immunohistochemically methods including detection of CD171- and CD3-positive cells and quantification of CD3+ cells in primary retinoblastoma tissue sections. (DOCX 20 kb) [file 12885_2019_6131_MOESM2_ESM.docx]

**Supplementary Method Description**

**Immunohistochemistry**. CD171 and CD3 were immunohistochemically detected in formalin-fixed, paraffin-embedded retinoblastoma sections on the VENTANA Benchmark XT automated staining instrument (Ventana Medical Systems, Tucson, AZ, USA) was used according to manufacturer's instructions. Slides were de-paraffinized in EZ prep solution for 30 minutes at 75°C, and antigen retrieval was performed in CC1 solution (Ventana Medical Systems) for 60 minutes at 95°C. Antibodies against CD171 (UJ127.11 clone, diluted 1:100, Thermo Scientific, Waltham, MS, USA) and CD3 (cat #A0452, diluted 1:100, Agilent, Santa Clara, CA, USA) were applied, and developed using the iVIEW^TM^ DAB Detection Kit (cat #760-091, Ventana Medical Systems). Two experienced neuropathologists independently evaluated CD171 staining intensity with concurrence. Counterstaining of the tumor sections was performed with hematoxylin for 4 minutes. CD3^+^ lymphocyte (T cell) infiltration into retrospective retinoblastoma tissue sections was assessed using the Stereo Investigator® Software system, including the Stereo Investigator® software (version 11.09 64bit, MBF Bioscience, Williston, VT, USA), an Olympus microscope BX53, the QImaging® camera COLOR 12 BIT and a MAC 6000 stage controller (Ludl Electronic Products Ltd., Hawthorne, NY, USA). The software randomly selected tissue areas, outlined at low magnification (4x objective lens). In this area, the software systematically positioned 400 × 400µm grids. Within these grids, T cells located in the two-dimensional optical dissector (85 × 85µm) were then counted at a higher magnification through the 20× objective lens at regularly pre-determined positions of the grid.
